# Porphyrins with combinations of 4-carboxyphenyl and 4-hydroxyphenyl substituents in *meso*-positions as anti-HIV-1 agents

**DOI:** 10.1038/s41598-024-60728-w

**Published:** 2024-05-01

**Authors:** Debdulal Sharma, Aradhana Singh, Sanaullah Safi, Ritu Gaur, Devashish Sengupta

**Affiliations:** 1 Department of Chemistry, Assam University, Silchar, Assam 788011 India; 2 Faculty of Life Sciences and Biotechnology, South Asian University, New Delhi, 110068 India

**Keywords:** Porphyrins, Photodynamic therapy, Photoinactivation of viruses, Photosensitizers, Biological techniques, Biotechnology, Chemical biology, Drug discovery, Molecular biology, Diseases, Chemistry

## Abstract

A series of 4-carboxyphenyl/4-hydroxyphenyl *meso*-substituted porphyrins were synthesized, purified, and characterized. The compounds exhibited anti-HIV-1 activities, in vitro, under both non-photodynamic (non-PDT) and photodynamic (PDT) conditions. Specifically, the porphyrins inhibited HIV-1 virus entry, with **c-PB**_**2**_**(OH)**_**2**_ and **PB(OH)**_**3**_ showing significant anti-HIV-1 activity. All of the porphyrins inhibited HIV-1 subtype B and C virus entry under PDT conditions. Our study demonstrated that the compounds bearing combinations of 4-carboxyphenyl/4-hydroxyphenyl moieties were not toxic even at higher concentrations, as compared to the reference porphyrins 5,10,15,20-tetra-(4-carboxyphenyl)porphyrin (**TCPP**) and 5,10,15,20-tetra-(4-hydroxyphenyl)porphyrin (**THPP**), under PDT conditions. This study underscores the promising potential of these compounds as HIV entry inhibitors in both non-PDT and PDT scenarios.

## Introduction

The human immunodeficiency virus (HIV), responsible for acquired immunodeficiency syndrome (AIDS), poses a formidable global health challenge, necessitating the exploration of innovative approaches to combat its spread and impact^1–3^. This has led to an exploration of porphyrins as agents that could disrupt various stages of the HIV life cycle, presenting a new avenue for antiviral drug development^4–10^. Porphyrins possess a unique molecular architecture that translates to remarkable photophysical properties and concomitant bio-compatibility allowing them to interact with biological macromolecules, such as proteins and nucleic acids, enabling diverse biological activities^11–15^. Researchers have increasingly focused on exploiting the interactions between porphyrins and viral components to inhibit crucial steps in the HIV life cycle, ranging from viral entry to replication and maturation^4–10^. Depending upon the overall charge of the porphyrin macrocycle, the mode of interaction with HIV and virucidal action on the virus differs from porphyrin to porphyrin^4–8,16^. Porphyrins act through RT inhibition^7,17–19^, HIV protease inhibition^20–22^ or by blocking the interaction with the V3 loop of gp120 glycoprotein^6,23^. In particular, anionic carboxylated or sulfonated porphyrins^24^ having a net negative charge were able to interact with the positively charged C5 region of the V3 loop of gp120 glycoprotein, thereby preventing the entry of HIV into the interacting cells. Additional photodamages to gp120 have also been reported^24^ post-irradiation with light. Notably *meso*-tetracarboxyphenyl porphyrin (**TCPP**) and its derivatives have been successful at inactivating HIV, with varying degrees of success^23^. Photodynamic treatment (PDT) is a clinically approved, non-invasive therapeutic method that targets cancers and microbial infections using a light source and a photosensitizer(PS). The process comprises administering a photosensitizer followed by irradiation at a wavelength that corresponds to the sensitizer’s absorbance band. The PS when activated by light at a specified wavelength, combines with molecular oxygen to produce reactive oxygen species in the target tissue, causing cell death^25^. **TCPP** is an excellent PS for cPDT^26–28^ and aPDT, per se, and in combination with NPs such as gold^29,30^, silica-coated magnetic NPs, graphene oxide quantum dots, and others^27,31,32^. Reports have also indicated a higher capability of cellular uptake. Along similar lines, porphyrin derivatives bearing *meso*-4-(hydroxyphenyl) moieties have been particularly useful. S_N_2 reaction at the –OH function with synthetic constructs bearing specific functional units enables the attachment of biologically potent molecules to the porphyrin unit, defining therapeutic outcomes. Derivatives of *meso*-(4-hydroxyphenyl)porphyrins standalone, or in combination with different electron-donating or electron-withdrawing group including fullerenes or conjugated with nanoparticles^33–35^ have shown enhanced photobiological outcomes in PDT. Several publications have reported the anti-HIV effects of substituted hydroxyphenyl porphyrins^4^. However, to the best of our knowledge, a systematic evaluation of the anti-HIV effects of porphyrins bearing a combination of carboxyphenyl and hydroxyphenyl moieties has not been reported.

Porphyrins are widely used in PDT due to their low toxicity, broad action, and low cost. They are highly effective in selectively targeting infected cells. The possibility of resistance development and genetic changes in infected people is negligible. The photo-inactivation of HIV-1 clinical variants, resistant variants, and HIV-2 variants by the porphyrins have also been reported^36,37^. Porphyrins may be a beneficial treatment for HIV-infected persons with high viral loads when used under PDT settings.

In the present publication, the authors have reported the synthesis, isolation, and characterization of an entire series of 4-carboxyphenyl/4-hydroxyphenyl *meso*-substituted porphyrins. The photophysical properties of the compounds were studied and several biological assays were carried out to determine the anti-HIV 1 efficacy of the compounds. The primary objective of the research outlined in this manuscript is to investigate the Structure–Activity Relationship (SAR) concerning the variations in *meso*-substituted combinations of 4-carboxyphenyl and 4-hydroxyphenyl in terms of their photobiological relevance as potential anti-HIV agents. Specifically, the positioning and function of carboxyphenyl and hydroxyphenyl groups, which play crucial roles in anti-HIV activities, significantly influence the photobiological relevance of porphyrins.

## Results and discussion

### Synthesis, isolation and characterization

The target water-soluble porphyrins and their precursors were synthesized through a multi-step synthetic pathway as outlined in Figure S1. The initial synthetic step involved a one-pot three-component reaction involving methyl-4-formylbenzoate (3.2 eq), 4-hydroxybenzaldehyde (1.8 eq) and pyrrole (4 eq) resulting in the formation of the A_4_, A_3_B, cis-A_2_B_2_, trans-A_2_B_2_, AB_3_ and B_4_ porphyrins 5,10,15,20-tetra-(4-methoxycarbonylphenyl)porphyrin (**PBe**_**4**_), 5,10,15-tri-(4-methoxycarbonylphenyl)-20-(4-hydroxyphenyl) porphyrin (**PBe**_**3**_**OH**), 5,10-di-(4-methoxycarbonylphenyl)-15,20-di-(4-hydroxyphenyl) porphyrin (**c-PBe**_**2**_**(OH)**_**2**_), 5,15-di-(4-methoxycarbonylphenyl)-10,20-di-(4-hydroxyphenyl) porphyrin (**t-PBe**_**2**_**(OH)**_**2**_) and 5-(4-methoxycarbonylphenyl)-10,15,20-tri-(4-hydroxyphenyl) porphyrin (**PBe(OH)**_**3**_) and 5,10,15,20-tetra-(4-hydroxyphenyl)porphyrin (**THPP**) respectively. The stoichiometry was chosen to obtain a higher yield of the A_3_B porphyrin. While TLC indicated the formation of all six porphyrins, isolation and purification of only **PBe**_**4**_, **PBe**_**3**_**OH**, **c-PBe**_**2**_**(OH)**_**2**_, **t-PBe**_**2**_**(OH)**_**2**_ and **PBe(OH)**_**3**_ was possible with appreciable yields. The compounds were isolated through gravity percolation column chromatography, silica gel 60–200 mesh was used as the stationary phase, while the mobile phase employed was either DCM or differing percentages (1–5%) of MeOH in DCM. Wherever required, column chromatography was run multiple times to obtain pure compounds. **THPP,** with four highly polar *meso*-hydroxyphenyl groups, did not elute out in a significant amount. It was synthesized separately as per a previously reported protocol^38^. The yield of the macrocycles **PBe**_**4**_, **PBe**_**3**_**OH**, **c-PBe**_**2**_**(OH)**_**2**_, **t-PBe**_**2**_**(OH)**_**2**_ and **PBe(OH)**_**3**_ were calculated to be 8%, 8%, 6%, 2% and 7% respectively. The isolated porphyrins were characterized by ^1^HNMR, ^13^CNMR, MALDI-TOF, UV–Vis, Emission and Fluorescence Lifetime spectroscopic techniques, whichever is applicable. The ^1^HNMR and MALDI-TOF data are fundamental for differentiation between the isolated porphyrins. The effect of the porphyrin ring diamagnetic anisotropy results in a large spread of ^1^HNMR resonances with the inner NH resonances being shifted to around –2.6 ppm, whereas the *meso*-substituted protons are shifted further downfield with, the deshielding being higher for *meso*-substituents with electron withdrawing groups, the β-pyrrolic protons at the periphery of the macrocyclic ring are also downshifted. The observed β-pyrrole splitting patterns (Table-S1) for the synthesized compounds are consistent with AB_3_/AB_3_, cis-A_2_B_2_, trans-ABAB systems as reported in literature. As can be seen in the ^1^HNMR spectral data (Figures S4, S6–S8, SI), the pyrrolic protons are split into doublet/singlet/doublet, doublet/singlet/singlet/doublet and doublet/doublet corresponding to the differing *meso*-substitution patterns. The porphyrins consistently show a negative peak at the far right, beyond the signal for TMS; the specific peak can be attributed to the highly shielded inner NH protons. Apart from that, the -OCH_3_ peak is consistently seen around 4.1 ppm in the recorded spectral data. The aromatic phenyl ring protons resonated between 8.4 and 7.4 ppm. The multiplicity, proton integration and spin–spin coupling constant for each compound is summarized in Table S1. The ^1^HNMR spectral data and the mass values (Figure S13–S16, SI) obtained through MALDI-TOF spectroscopy are indicative of the purity of the synthesized porphyrin macrocycles.

The UV–Vis spectra of **PBe**_**3**_**OH**, **c-PBe**_**2**_**(OH)**_**2**_, **t-PBe**_**2**_**(OH)**_**2**_ and **PBe(OH)**_**3**_, recorded in DMF conform to the D_2_h micro symmetry in accordance with the Four-Orbital Model suggested by Gouterman. The spectra of the porphyrin macrocycles consistently show an intense absorption near 420 nm (Soret Band) and much less intense Q-band absorption between 500 and 700 nm (Table S2). Interestingly, with an increase in the number of more polar *meso*-hydroxyphenyl moieties, a slight red shift in Soret band absorption is observed (Figure S2A). The Q-bands show subtle differences indicating variations in the skeletal structure of the compounds; however, no specific trend can be attributed to the structural differences. The emission spectra of the porphyrins **PBe**_**4**_, **c-PBe**_**2**_**(OH)**_**2**_, and **t-PBe**_**2**_**(OH)**_**2**_ and recorded in DMF exhibit twin emission peaks (Table S2, Figure S2B) corresponding to the Q(0,0) and Q(0,1) emission band, expected for a free base system. In addition to the twin emissions, **PBe**_**3**_**OH** and **PBe(OH)**_**3**_, however, exhibit a low-intensity emission band centred at 645 and 616 nm respectively, this could be attributed to a Q (1,0) transition. The solutions were excited at the absorption maxima of their respective Soret bands.

**Figure 1:**
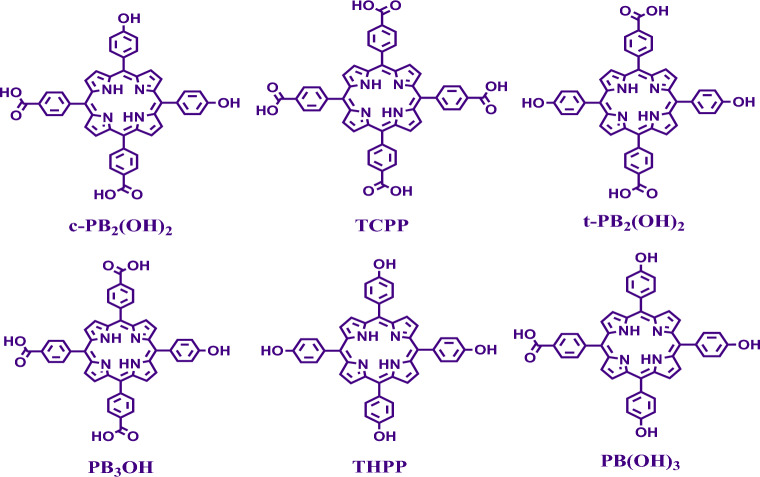
Water-soluble porphyrins prepared having combinations of *meso*-(4-carboxyphenyl) and *meso*-(4-hydroxyphenyl) groups.

The compounds **PBe**_**4**_, **PBe**_**3**_**OH, c-PBe**_**2**_**(OH)**_**2**_, **t-PBe**_**2**_**(OH)**_**2**_, **PBe(OH)**_**3**_ were hydrolysed (Figure S1) to obtain tetra-(4-carboxyphenyl)porphyrin **(TCPP)**, 5,10,15-tri-(4-carboxyphenyl)-20-(4-hydroxyphenyl) porphyrin (**PB**_**3**_**OH**), 5,10-di-(4- carboxyphenyl)-15,20-di-(4-hydroxyphenyl) porphyrin (**c-PB**_**2**_**(OH)**_**2**_), 5,15-di-(4- carboxyphenyl)-10,20-di-(4-hydroxyphenyl) porphyrin (**t-PB**_**2**_**(OH)**_**2**_), and 5-(4- carboxyphenyl)-10,15,20-tri-(4-hydroxyphenyl) porphyrin (**PB(OH)**_**3**_), bearing combinations of *meso*-(4-hydroxyphenyl) and *meso*-(4-carboxyphenyl) moieties (Fig. 1). In brief, the weighed-out amount of the ester precursors was treated with crushed NaOH in DMF, for 30 min, followed by the addition of water. The pH of the reaction mixture was adjusted to 4.5 when the desired product precipitated out. The compounds were recovered by centrifugation. **TCPP**, **PB**_**3**_**OH**, **c-PB**_**2**_**(OH)**_**2**_, **t-PB**_**2**_**(OH)**_**2**_** and PB(OH)**_**3**_ were obtained in 78%, 85%, 98%, 78%, and 82% yield, respectively. It may be mentioned here that the compounds **PB**_**3**_**OH, PB(OH)**_**3**_, **t-PB**_**2**_**(OH)**_**2**_ have been reported earlier with different synthetic protocol^39–41^.

The detailed analysis of ^1^HNMR spectral plots (Figures S9–S12, SI) of **PB**_**3**_**OH**, **c-PB**_**2**_**(OH)**_**2**_**, t-PB**_**2**_**(OH)**_**2**_ and **PB(OH)**_**3**_ is shown in Table S3. The data conforms to the splitting pattern expected of a cis-A_2_B_2_, trans-A_2_B_2_ and AB_3_ system. The β-pyrrole protons resonate as a multiplet centred at 8.88 ppm for **c-PB**_**2**_**(OH)**_**2**_, for **t-PB**_**2**_**(OH)**_**2**_ and **PB(OH)**_**3**_ however, the protons are split into two doublets (centered at 8.81 and 8.91 ppm) and a doublet-singlet-doublet (at 8.79 (*J* = 2.4 Hz,)-8.82 (s)-9.95 (*J* = 6.1 Hz) ppm) respectively. The aromatic phenyl protons of the compounds resonated between 7.0 and 8.5 ppm as outlined in Table S3.

The peak for the labile protons of -COOH and -OH groups could not be detected for compounds **c-PB**_**2**_**(OH)**_**2**_ and **PB(OH)**_**3**_, possibly as a result of deuterium exchange. All three compounds **c-PB**_**2**_**(OH)**_**2**_**, t-PB**_**2**_**(OH)**_**2**_, and **PB(OH)**_**3**_ show the inner pyrrolic proton resonances at − 2.87, − 2.91 and − 2.88 ppm respectively. MALDI-TOF data lent further support in favour of the molecular structure of the compounds, the theoretical values of mass conform well with the experimental values (Figures S17–S20, SI). The UV–Vis and emission spectral data for **c-PB**_**2**_**(OH)**_**2**_**, t-PB**_**2**_**(OH)**_**2**_**, PB(OH)**_**3**_, **PB**_**3**_**OH** and **TCPP**, corresponding extinction coefficient and Stokes shifts are included in detail in Table S4. The UV–Vis spectral plot (Figure S3A) of the compounds exhibits the intense Soret band and Q-bands expected of the porphyrinic system. The Soret band maxima of **t-PB**_**2**_**(OH)**_**2**_ is red shifted to 426 nm as compared to that of the other compounds. Not-so-significant redshifts in the Soret band maxima are also seen in **PB**_**3**_**OH**, **c-PB**_**2**_**(OH))**_**2**_, and **PB(OH)**_**3**_ as compared to that of **TCPP**. The Q-band absorptions show progressive redshifts as the number of *meso*-hydroxyphenyl substituents increases in the porphyrin macrocycle, the red shift however does not follow any regular pattern. The Q_x_(0,0) band of **c-PB**_**2**_**(OH)**_**2**_**,** and **PB(OH)**_**3**_ shows strong redshift with absorption values of 690 and 684 nm respectively. It may be because the electron-donating capability of the hydroxyphenyl moieties boosts the aromatic ring current of the porphyrin macrocycle thereby bringing about a change in the (π, π*) transitions.

The emission spectra (Figure S3B and Table S4) of **PB**_**3**_**OH** and **TCPP** exhibit twin emission peaks corresponding to Q(0,0) and Q(0,1) transitions. However, for **c-PB**_**2**_**(OH))**_**2**_, **t-PB**_**2**_**(OH))**_**2**_ and **PB(OH)**_**3**_, triple emission peaks were observed at 612, 652, 710 nm, 612, 653, 708 nm and 614, 657, 710 nm corresponding to Q(1,0), Q(0,0) and Q(0,1) bands^42^.

The fluorescence quantum yields (Φf) of the target hydrophilic compounds (Table 1) were determined at the same concentration (10 µM) using **TCPP** in ethanol (Φf = 0.044) as the standard^43^. The following equation was used to determine the Φf values:

$$\varPhi_{{f}} = \frac{{{I}}\left( {{1} - {10}^{{{-A}}}} \right)_{{{std}}} \eta^{2}} {{{I_{std}}}\left( {{1} - {10} ^{{-A}}} \right){\eta_{std}^{2}}}$$

where Φf, I, A, and η are the fluorescence quantum yield, integral area of fluorescence, absorbance in λ_exc_, and refractive index of the selected solvents (H_2_O = 1.333 and Ethanol = 1.3614). The subscript “std” refers to the standard molecule.

**Table 1:** Fluorescence Quantum yield of **c-PB**_**2**_**(OH)**_**2**_, **t-PB**_**2**_**(OH)**_**2**_, **PB(OH)**_**3**_, **PB**_**3**_**OH** and **TCPP** recorded in water at a concentration of 10 µM.

	Fluorescence quantum yield
Compound	Φf
**PB**_**3**_**OH**	0.044
**c-PB**_**2**_**(OH)**_**2**_	0.025
**t-PB**_**2**_**(OH)**_**2**_	0.003
**PB(OH)**_**3**_	0.001
**TCPP**	0.059

The compound TCPP exhibited the highest Φf value, and as the number of carboxyphenyl groups within the porphyrin moiety decreased, the Φf values also showed a progressive decrease. Fluorescence quantum yield of 0.012 was reported for **PB(OH)**_**3**_ and **t-PB**_**2**_**(OH)**_**2**_ earlier, in a benzene solution using TPP (Φf = 0.11) as the standard^39^. The difference in the Φf's can be attributed to the polarity difference between the solvents used for measuring the spectral data^44^.

### Anti-HIV studies

In this study, the carboxyphenyl porphyrins tested against HIV-1 virus were **TCPP, THPP, PB**_**3**_**OH, c-PB**_**2**_**(OH)**_**2**_**,** **t-PB**_**2**_**(OH)**_**2**_, and **PB(OH)**_**3**_. The reference controls were THPP (tetra-hydroxyphenyl porphyrin) and TCPP (tetra-carboxyphenyl porphyrin). Enfuvirtide (T20), an FDA-approved fusion and entry inhibitor, was used as the positive control^45^. The anti-viral studies for these novel compounds were conducted under non-photodynamic (non-PDT) and photodynamic (PDT) conditions.

For the photodynamic therapy, the cells were incubated with serial dilutions of the test compounds before being irradiated for 45 min in an irradiation box. The irradiation box had the dimensions of length 20", width 6", and height 8", and it was equipped with two Philips Essential Master PL-L 36W/865/4P linear, compact fluorescent lamps. The surface of a 96-well microplate was deemed to be 15 cm from the light source. 250 J cm^−2^ of light was determined to be present at the surface.

#### Carboxyphenyl porphyrins were not toxic to the cells under non-PDT and PDT conditions

The effect of the carboxyphenyl porphyrins on the cell viability was tested on HEK293T and TZM-bl cell lines under non-PDT and PDT conditions by employing the Cell-Titre blue Assay as described in the methods section. The compounds were tested at the concentration ranging from 0.1 to 50 µM. We observed that none of the porphyrins were cytotoxic under non-PDT conditions with a CC_50_ value greater than 30 µM. **PB**_**3**_**OH, TCPP**, and **THPP** had a CC_50_ value lower than 5 µM in the PDT conditions (Table S5). They were toxic to the cells at higher concentrations. The compounds **c-PB**_**2**_**(OH)**_**2**_, **t-PB**_**2**_**(OH)**_**2**,_ and **PB(OH)**_**3**_ were non-toxic to the cells even at the higher concentrations (CC_50_ > 30 µM). Following this assay, the non-toxic concentrations of the porphyrins (up to 5 µM for non-PDT and 500 nM for PDT conditions) were used to perform the anti-viral studies.

#### Carboxyphenyl porphyrins reduced HIV-1 virus infectivity under non-PDT conditions

The antiviral activity of the carboxyphenyl porphyrins was assessed by determining their effect on HIV-1 gene expression and virus release.

HEK-293 T cells were transfected with plasmid DNA containing either the HIV-1 subtype B NL4-3 virus or the HIV-1 subtype C K3016 virus.

The cells were then treated with the test compounds at a concentration ranging from 500 nM to 5 µM and incubated at 37 °C for 24 h. HIV-1 p24 ELISA was used to determine the amount of virus released in the supernatant. Both the cells and virus lysates were immunoblotted with the total HIV IgG antibody. The virus release efficiency (%VRE) in the presence of test compounds was compared to the negative control, 5% DMSO in water (-). We observed that incubating cells with the test compounds did not affect viral gene expression and the subsequent virus release from the cells for both subtypes compared to the control (Figure S22). A TZM-bl cell-based single-cycle infectivity assay was used to assess the infectivity of the viruses produced in the presence or absence of porphyrins^46,47^.

**Figure 2:**
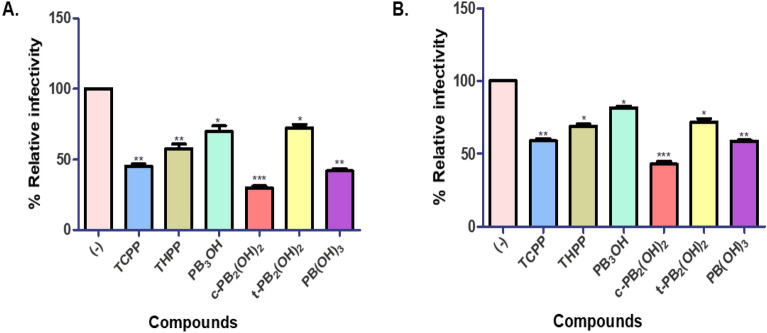
The carboxyphenyl porphyrins reduced the HIV-1 virus infectivity under non-PDT conditions Relative infectivity of (**A**) HIV-1 subtype B NL4-3 viruses are produced in the presence or absence of 5 µM carboxyphenyl porphyrins in TZM-bl cells. (**B**) HIV-1 subtype C K3016 viruses are produced in the presence or absence of 5 µM porphyrins in TZM-bl cells. Quantitative data for levels of infectivity relative to 5% DMSO in water (-) is shown (n = 3). Error bars indicate standard deviations from three independent experiments. *p-value < 0.05**p-value < 0.01***p value < 0.001: student’s t-test.

The expression of a luciferase reporter gene under the control of the HIV-1 LTR promoter is used in this assay, which is regarded as a sensitive and quantitative marker of virus infection. The infectivity of HIV-1 subtype B NL4-3 virus or HIV-1 subtype C K3016 virus produced in the presence of 5% DMSO in water (-), in the absence of the carboxyphenyl porphyrins, served as the treatment control for the experiment. The porphyrins **c-PB**_**2**_**(OH)**_**2**_ and **PB(OH)**_**3**_ reduced the infectivity of produced HIV-1 subtype B NL4-3 virus by more than 60%. The reference compounds **TCPP** rendered about 50% of the viruses non-infectious followed by **THPP**, and **PB**_**3**_**OH** (Fig. 2A). Similarly, **c-PB**_**2**_**(OH)**_**2**_ reduced the infectivity of the HIV-1 subtype C K3016 virus by 50%, followed by **TCPP**, **PB(OH)**_**3**_ and THPP. (Fig. 2B). These results indicate that compared to other carboxyphenyl porphyrins, the cis-conformation in **c-PB**_**2**_**(OH)**_**2**_ and the presence of three hydroxyl groups in **PB(OH)**_**3**_ were most effective in reducing the infectivity of HIV-1 subtype B and subtype C virus.

#### Carboxyphenyl porphyrins strongly restricted HIV-1 entry and infection under non-PDT conditions

The impact of carboxyphenyl derivatives on virus entry, a preliminary stage of the HIV-1 life cycle, was examined to evaluate their anti-HIV-1 activity. TZM-bl cells were used for entry inhibition assays as per the methods section. Briefly, TZM-bl cells were infected with 10 ng of HIV-1 p24 equivalent NL4-3 or K3016 virus, followed by the addition of different concentrations of compounds (100 nM–5 µM) during virus infection as described in methods. After washing, the infected cells were incubated at 37 °C for 48 h. After 48 h, relative luciferase activity was assessed and compared to the control (-). In this case, "control" refers to HIV-1 subtype B NL4-3 or subtype C K3016 virus-infected cells not exposed to test compounds. Enfuvirtide (T20), an HIV fusion inhibitor, was employed as a positive control.

**Figure 3:**
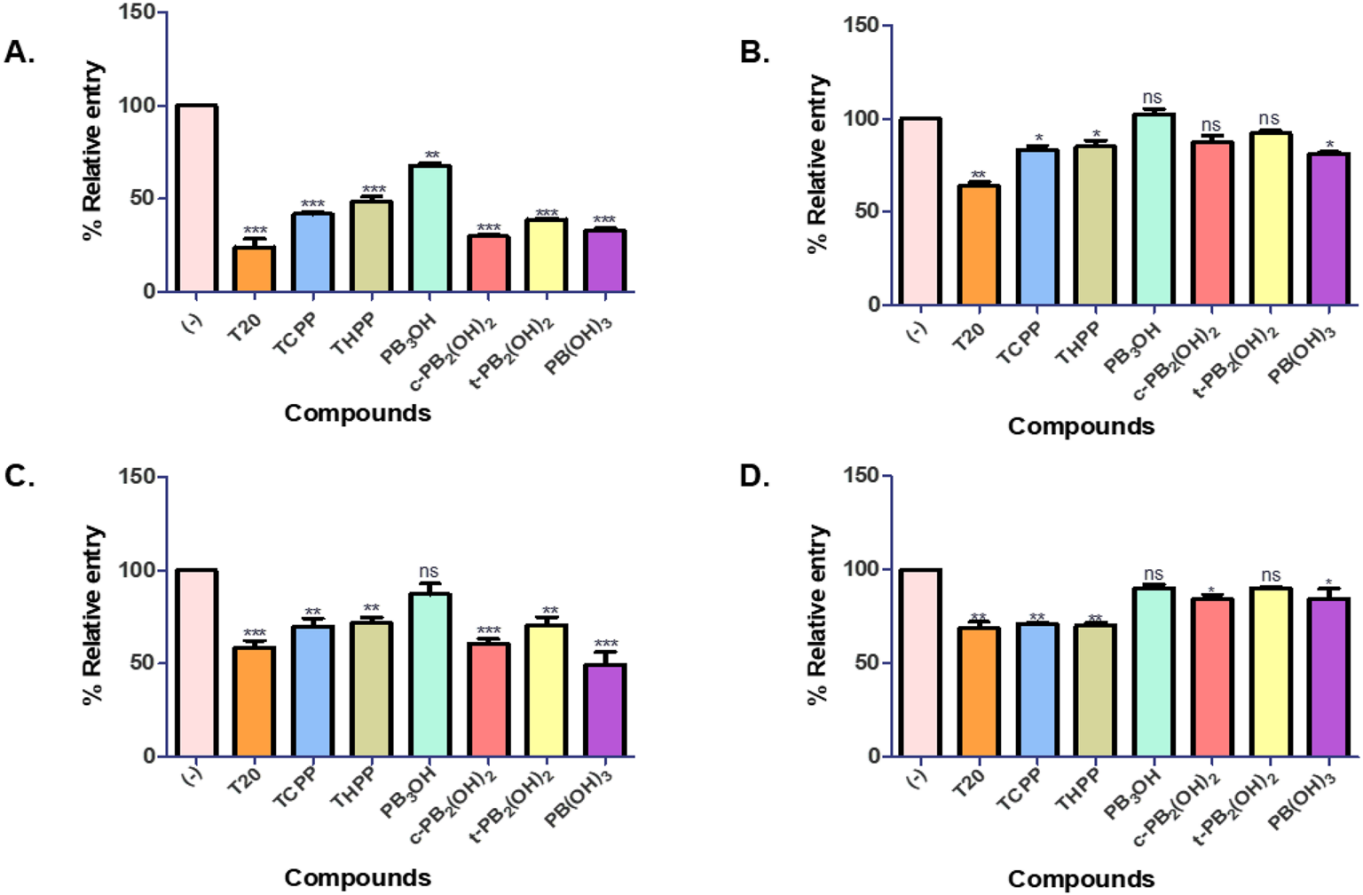
Entry inhibition assays for HIV-1 subtype B NL4-3 virus and HIV-1 subtype C K3016 virus under non-PDT conditions: (**A**) Compounds were added during virus infection of TZM-bl cells with HIV-1 p24 normalized subtype B NL4-3 virus. (**B**) Compounds were added post-infection with the HIV-1 subtype B NL4-3 virus. (**C**) Compounds were added during virus infection of cells with the HIV-1 p24 normalized subtype C K3016 virus. (**D**) Compounds were added post-infection with the HIV-1 subtype C K3016 virus (n = 3). 0.5 µM T20 was used as a positive control. Quantitative data for virus entry relative to the 5% DMSO in the water (-) control sample is shown (n = 3). Error bars indicate standard deviations (n = 3). *p-value < 0.05**p-value < 0.01***p value < 0.001: student’s t-test.

We observed that these carboxyphenyl porphyrins inhibited the virus entry in a dose-dependent manner. At 5 µM maximal concentration, compounds **c-PB**_**2**_**(OH)**_**2**_ and **PB(OH)**_**3**_ significantly reduced the entry of HIV-1 subtype B NL4-3 virus by **70%,** followed by a **60%** entry inhibition by **t-PB**_**2**_**(OH)**_**2**_. The positive control, **T20**, prevented the virus entry by **80%** at 0.5 µM concentration. The reference compounds **TCPP** and **THPP** blocked the virus entry by nearly 60%, while the precursor **PB**_**3**_**OH** showed a lower inhibition of 30% (Fig. 3A). The EC_50_ values for **c-PB**_**2**_**(OH)**_**2**_, **t-PB**_**2**_**(OH)**_**2**_, and **PB(OH)**_**3**_ were determined to be 2.622 µM,4.098 µM, and 2.44 µM, respectively. Similarly, in the case of HIV-1 subtype C K3016 virus, compounds **PB(OH)**_**3,**_ and **c-PB**_**2**_**(OH)**_**2**_, **t-PB**_**2**_**(OH)**_**2**_ prevented its entry by 50%,40% and 30%, respectively. The positive control, **T20**, and the reference compounds **TCPP** and **THPP** restricted the virus entry by 40% and 30%, respectively (Fig. 3C). For the HIV-1 subtype C K3016 virus, the EC_50_ value of these porphyrins was found to be more than 5 µM. Also, these compounds effectively block the entry of the HIV-1 subtype B NL4-3 virus compared to the HIV-1 subtype C K3016 virus. Next, we wanted to study the effect of these compounds on the post-entry stages of the virus life cycle. For this, TZM-bl cells were infected with the HIV-1 subtype B NL4-3 virus or HIV-1 subtype C K3016 virus for 2 h. The infected cells were washed and incubated for 48 h with the carboxyphenyl derivatives. We observed that none of the compounds could effectively reduce HIV-1 subtype B NL4-3 or HIV-1 subtype C K3016 virus production post-entry to the cells. (Fig. 3B,D). These results suggested the role of carboxyphenyl derivatives in inhibiting the early stages of HIV-1 entry under non-PDT conditions.

#### Carboxyphenyl porphyrins restricted the virus entry in T cells

Carboxyphenyl porphyrins were also examined for their ability to reduce HIV-1 subtype B NL4-3 or subtype C K3016 virus entry in HutR5 cells (Human T-cell line). These cells were infected with 10 ng of HIV-1 p24 equivalent subtype B NL4-3 or subtype C K3016 virus for 2 h at 37 °C in the presence (5 µM) or absence of test compounds. HIV-1 p24 ELISA was used to quantify the virus. When added during infection, only the derivative **c-PB**_**2**_**(OH)**_**2**_ could inhibit the entry of HIV-1 subtype NL4-3 virus and subtype C K3016 virus by 50% and 30%, respectively (Figure S23a,c). No reduction in virus infection was observed in both HIV-1 subtypes when the compounds were added post-virus entry in the HutR5 cells (Figure S23b,d). Hence, the carboxyphenyl porphyrins also inhibited virus entry in T cells when introduced early in the infection.

#### Carboxyphenyl derivatives did not bind cellular CD4 receptors or co-receptors

The HIV-1 virus infects the host cells through specific binding of HIV-1 envelope (Env) glycoproteins gp120 with cellular CD4 receptors and chemokine co-receptors CXCR4 or CCR5^48^. It prompts the viral gp41 transmembrane protein to mediate the fusion of the viral and cell membranes, enabling the virus to enter and deliver its genetic material inside the cells. We hypothesized that carboxyphenyl porphyrins inhibited the early stages of virus entry. We further assessed whether this inhibitory effect of these compounds relied on their specific interactions with viral envelope proteins or host-cell receptors/co-receptors. TZM-bl cells were pre-incubated with the compounds before infection with HIV-1 subtype B NL4-3 or subtype C K3016 virus for 2 h at 37 °C. It ensured that all the receptors were saturated before adding the virus. We observed that pre-incubation of cells with carboxyphenyl porphyrins did not restrict virus infection (Figure S24a,b). The lack of entry inhibition of the HIV-1 subtype B NL4-3 or subtype C K3016 virus in the pre-treated TZM-bl cells suggested that these porphyrins do not interact with the host cell receptors or coreceptors. These results indicate that the carboxyphenyl porphyrins inhibit the virus entry in the target cells by possible interactions with the viral envelope proteins instead of host cell receptors and co-receptors.

#### Carboxyphenyl porphyrins were most effective in blocking the HIV-1 virus entry when added early during infection

**Figure 4:**
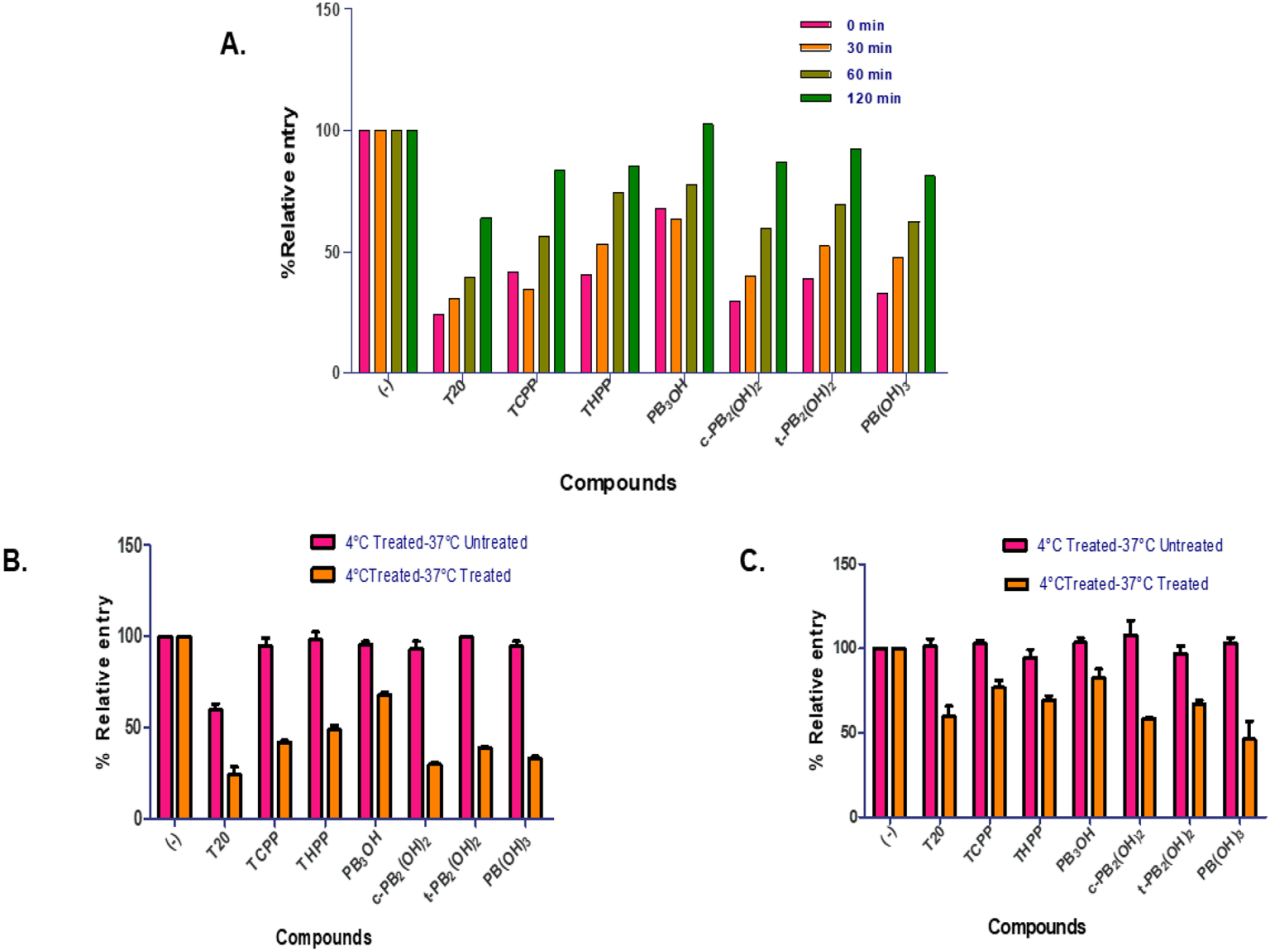
(**A**) Time of addition assay-TZM-bl cells were infected with the HIV-1 subtype B NL4-3 virus. The addition of carboxyphenyl porphyrins was delayed by 0 min, 30 min, 1 h, and 2 h. (**B**) Temperature arrest assay was performed at 4 °C and 37 °C. TZM-bl cells were infected with HIV-1 subtype B NL4-3 virus or (**c**). HIV-1 subtype C K3016 virus in the presence of compounds at 4 °C followed by washing and further incubation at 37 °C for 48 h in the presence or absence of the compounds. Quantitative data for virus entry relative to the 5% DMSO in the water (-) control sample is shown (n = 3). Error bars indicate standard deviations (n = 3).

To better understand the mode of action and the key targets, carboxyphenyl porphyrins were introduced to the host cells at different time points. It sought to establish how soon after the virus was added, the compounds may prevent its entry into the cells. 10 ng of p24 equivalent HIV-1 subtype B NL4-3 virus was used to infect TZM-bl cells. 5 µM of carboxyphenyl porphyrins were added to the cells at four-time points post-virus addition: 0 min, 30 min, 1 h, and 2 h. Here, control cells were the infected cells treated with 5% DMSO in water (-) without compounds. We observed that the extent of virus entry inhibition was time-dependent. As the addition of compounds progressed to the later time points, their ability to inhibit virus entry was impaired. It means that the carboxyphenyl porphyrins were most potent when added during the HIV-1 subtype B NL4-3 virus infection. (Fig. 4A). The compounds **c-PB**_**2**_**(OH)**_**2**_ and **PB(OH)**_**3**_ inhibited virus entry by nearly 70%, followed by 60% inhibition by **t-PB**_**2**_**(OH)**_**2**_ when combined with the virus. Delaying the addition of compounds for 30 min after virus addition resulted in 50–60% restriction in virus entry compared to 40% inhibition by **t-PB**_**2**_**(OH)**_**2**_. These results implied that the carboxyphenyl porphyrins prevented virus infection maximally when added during the early stages of virus entry.

#### Carboxyphenyl porphyrins operate as a post-binding inhibitor of HIV-1 entry

The interaction of viral gp120 with cellular CD4 receptors has been reported to occur at 4 °C, but the fusion of the viral and cell membranes requires 37 °C. We performed a temperature arrest assay at 4 °C and 37 °C in the presence or absence of carboxyphenyl porphyrins to determine whether they prevent the viral entry at the binding or post-binding stages. TZM-bl cells were infected with 10 ng of HIV-1 equivalent subtype B NL4-3 or subtype C K3016 virus at 4 °C in the presence or absence of the test compounds. The cells were washed and incubated at 37 °C for 48 h in the presence or absence of the compounds. The relative luciferase activity of infected cells was compared to those treated with 5% DMSO in water (-) without compounds (negative control). If the porphyrins prevented the virus’s attachment to cellular receptors at 4 °C, then incubating cells at 37 °C in their absence would prevent virus entry. However, our findings demonstrated that HIV-1 subtype B NL4-3 or subtype C K3016 viral entry was blocked substantially only when cells were incubated at 37 °C in the presence of the compounds. At 37 °C, **T20, c-PB**_**2**_**(OH)**_**2**_, and **PB(OH)**_**3**_ blocked the HIV-1 subtype B NL4-3 virus entry by 70%. **TCPP, THPP,** and **t-PB**_**2**_**(OH)**_**2**_ exhibited more than 50% entry inhibition, followed by 30% inhibition by **PB**_**3**_**OH** (Fig. 4B). The HIV-1 subtype C K3016 virus entry was inhibited by 50% by **PB(OH)**_**3**_. **T20 and c-PB**_**2**_**(OH)**_**2**_**, t-PB**_**2**_**(OH)**_**2**_** and THPP, TCPP and PB**_**3**_**OH** restricted the virus entry by nearly 40%, 30%, and 20%, respectively (Fig. 4C). At 4 °C, no inhibition of virus entry for any porphyrin in both the HIV-1 subtypes was observed. These findings revealed that porphyrins did not prevent the viral envelope glycoprotein gp120 from interacting or attaching to the cellular CD4 receptor. Hence, these carboxyphenyl derivatives were considerably more active during the post-binding events of the HIV-1 virus entry.

#### Carboxyphenyl porphyrins strongly restricted HIV-1 entry and infection under PDT conditions

**Figure 5:**
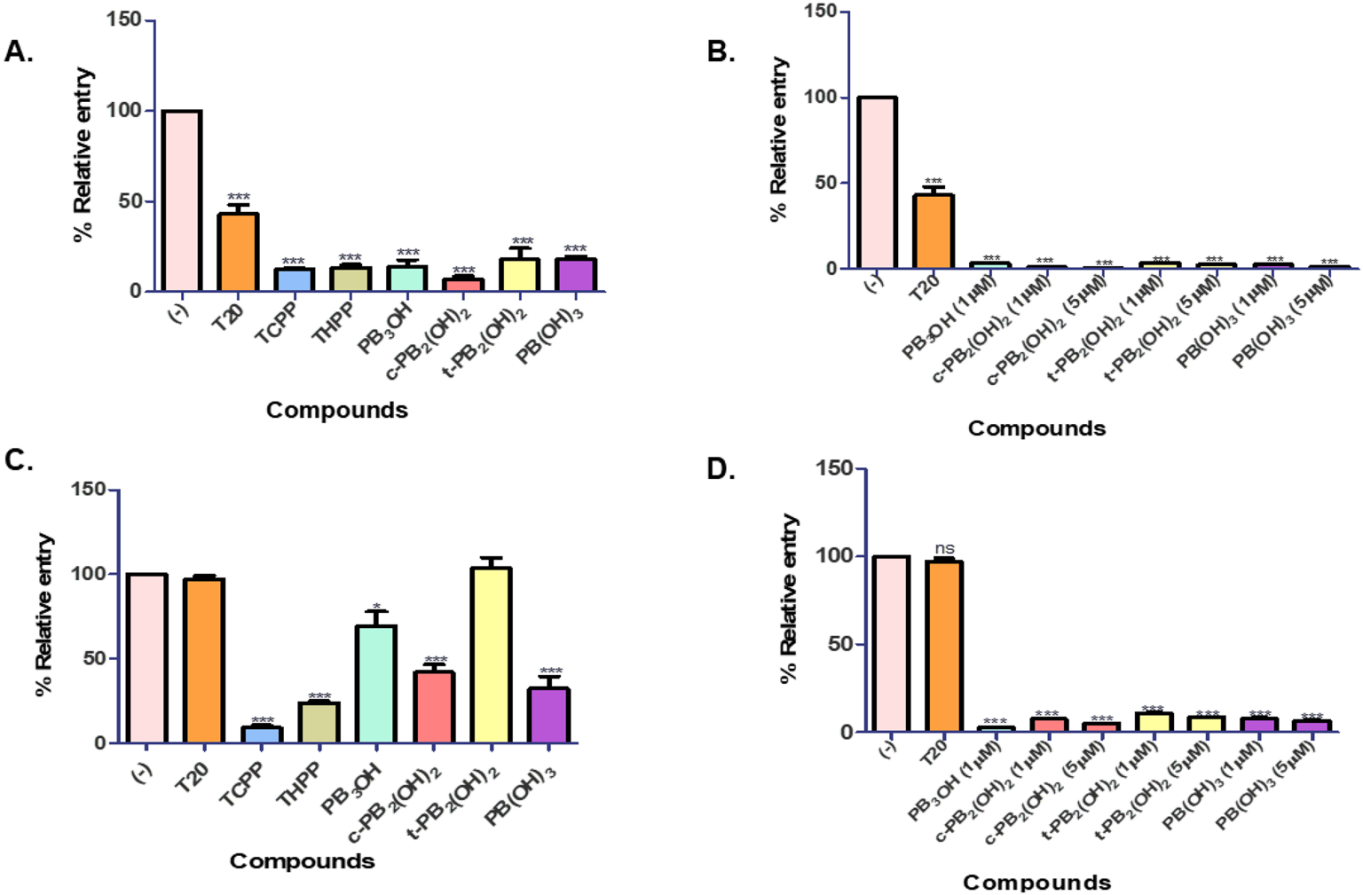
Entry inhibition assays for HIV-1 subtype B NL4-3 virus under PDT conditions: (**A**) HIV-1 subtype B NL4-3 virus pre-treated with the porphyrins at 500 nm concentration before infection in TZM-bl cells. (**B**) HIV-1 subtype B NL4-3 virus pre-treated with the compounds, PB_3_OH (1 µM), **c-PB**_**2**_**(OH)**_**2**_ (1 µM and 5 µM)_**,**_** t-PB**_**2**_**(OH)**_**2**_ (1 µM and 5 µM), and **PB(OH)**_**3**_ (1 µM and 5 µM) before infection in TZM-bl cells. (**C**) Porphyrins were added post-infection with HIV-1 subtype B NL4-3 virus in PDT conditions at 500 nM concentration. (**D**) Porphyrins were added post-infection HIV-1 subtype B NL4-3 virus infection in cells, PB_3_OH (1 µM), **c-PB**_**2**_**(OH)**_**2**_ (1 µM and 5 µM)_,_** t-PB**_**2**_**(OH)**_**2**_ (1 µM and 5 µM), and **PB(OH)**_**3**_ (1 µM and 5 µM). Quantitative data for virus entry relative to the 5% DMSO in the water (-) control sample is shown. Error bars indicate standard deviations (n = 3). *p-value < 0.05**p-value < 0.01***p value < 0.001: student’s t-test.

**Figure 6:**
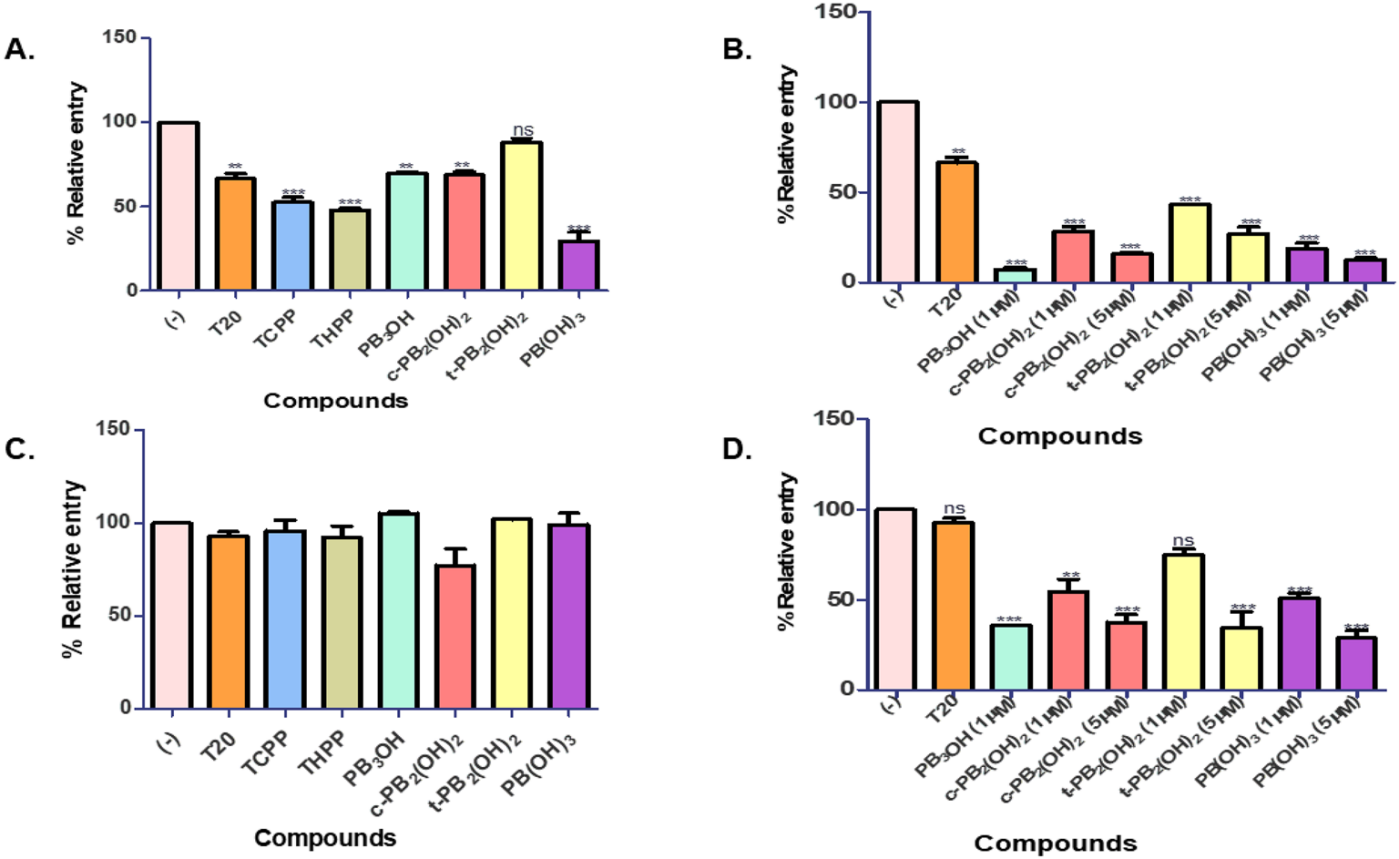
Entry inhibition assays for HIV-1 subtype C K3016 virus under PDT conditions: (**A**) HIV-1 subtype C K3016 virus was pre-treated with the porphyrins at 500 nM concentration and used to infect TZM-bl cells (n = 3). (**B**) HIV-1 K3106 virus was pre-treated with the compounds, PB_3_OH (1 µM), **c-PB**_**2**_**(OH)**_**2**_ (1 µM and 5 µM)_**,**_** t-PB**_**2**_**(OH)**_**2**_ (1 µM and 5 µM), and **PB(OH)**_**3**_ (1 µM and 5 µM), before infection in cells. (n = 3) (**C**) Porphyrins incubated post-infection in PDT conditions for 48 h at 500 nM concentration (n = 3) (**D**) Porphyrins incubated post-infection in PDT conditions, PB_3_OH (1 µM), **c-PB**_**2**_**(OH)**_**2**_ (1 µM and 5 µM)_**,**_** t-PB**_**2**_**(OH)**_**2**_ (1 µM and 5 µM), and **PB(OH)**_**3**_ (1 µM and 5 µM). Quantitative data for virus entry relative to the 5% DMSO in the water (-) control sample is shown. Error bars indicate standard deviations (n = 3). *p-value < 0.05**p-value < 0.01***p value < 0.001: student’s t-test.

We investigated the effect of test compounds on virus entry under PDT conditions, as stated in the methods. Under PDT conditions, 10 ng of HIV-1 p24 equivalent NL4-3 or K3016 virus was pre-incubated with the carboxyphenyl derivatives. The pre-treated virus was then utilized to infect TZM-bl cells. Infected cells were washed and cultured at 37 °C for 48 h. The relative luciferase activity was calculated concerning the control. The term "control" refers to virus-infected cells treated with 5% DMSO in water (-) in the absence of compounds. Enfuvirtide (T20) was utilized as a positive control. The carboxyphenyl porphyrins effectively prevented HIV-1 subtype B NL4-3 or subtype C K3016 viral entry at the higher concentrations in a dose-dependent manner at concentrations ranging from 10 nM to 5 µM. The maximum concentrations used for the compounds **TCPP, THPP** were 500 nM and for **PB**_**3**_**OH** 1 µM, because they were found to be cytotoxic at higher concentrations in PDT conditions. Under PDT conditions at 500 nM concentration, the reference porphyrin **TCPP** strongly restricted HIV-1 subtype B NL4-3 virus entry by 96%, followed by 90 to 95% inhibition by **c-PB**_**2**_**(OH)**_**2**,_ **PB**_**3**_**OH**, and **THPP.** More than 85% of virus entry inhibition was observed by the porphyrins **t-PB**_**2**_**(OH)**_**2**_** and PB(OH)**_**3**_ (Fig. 5A). The porphyrin **PB**_**3**_**OH** blocked the virus entry by 97% at 1 µM, followed by more than 98% virus entry inhibition by **c-PB**_**2**_**(OH)**_**2,**_** t-PB**_**2**_**(OH)**_**2**_** and PB(OH)**_**3**_ at 5 µM (Fig. 5B). These compounds showed anti-HIV activity in a dose-dependent manner. Post-infection, none of the porphyrins were able to inhibit the virus production at 500 nM concentration (Fig. 5C). However, at 1 µM concentration, **PB**_**3**_**OH** reduced the virus production by 95%. The porphyrins **c-PB**_**2**_**(OH)**_**2,**_** t-PB**_**2**_**(OH)**_**2**_** and PB(OH)**_**3**_ effectively blocked HIV-1 production by 90 to 95% at 5 µM. (Fig. 5D). The porphyrins exhibited EC_50_ value < 50 nM under PDT conditions for HIV-1 subtype B NL4-3 virus. The carboxyphenyl porphyrin **PB(OH)**_**3**_ inhibited HIV-1 subtype C K3016 virus entry by 70%, while the reference porphyrins **TCPP** and **THPP** inhibited entry by nearly 50% at 500 nM (Fig. 6A). At 1 µM, **PB**_**3**_**OH** restricted the virus entry by more than 90%, followed by more than 85% reduction at 5 µM for the compounds **c-PB**_**2**_**(OH)**_**2**_ and **PB(OH)**_**3**_ (Fig. 6B). The EC_50_ value of the compounds for HIV-1 subtype C virus was nearly 500 nM or more. The porphyrins were not active against HIV-1 subtype C virus post-infection at 500 nM under the PDT conditions (Fig. 6C). However, upon increasing the concentration to 1 µM for **PB**_**3**_**OH** and 5 µM for **c-PB**_**2**_**(OH)**_**2,**_** t-PB**_**2**_**(OH)**_**2**,_ and **PB(OH)**_**3**,_ the virus production was decreased by more than 60% and 80%, respectively (Fig. 6D).

## Discussion

This study aimed to test the carboxyphenyl porphyrins for their anti-HIV activity. These porphyrins did not exhibit cytotoxicity even at higher concentrations. They effectively restricted the HIV-1 virus entry to the cells under non-PDT and PDT conditions at low micromolar and nanomolar concentrations. Several porphyrin derivatives, notably *meso*-tetra (4-carboxyphenyl) porphyrin, have previously been described as HIV-1 replication inhibitors^23^. The carboxyphenyl porphyrins **c-PB**_**2**_**(OH)**_**2**_ and **PB(OH)**_**3**_ significantly limited the HIV-1 subtypes B and C virus entry. They also interfered with virus entry in Human T cells. The HIV-1 envelope protein consists of gp120 and gp41. Interaction between gp120 and CD4 receptors on T cells exposes conserved domains, including V3 and V1/V2 loops, crucial for co-receptor binding. Binding of gp120 to CCR5/CXCR4 induces conformational changes in both gp120 and gp41. This triggers extension of the gp41 fusion peptide and formation of a helical coiled-coil, facilitating viral and host membrane fusion^48^. The HIV entry inhibitors disrupt viral recruitment and cell membrane fusion, impeding the infection cycle. This strategy creates a barrier against virus entry, reduces latent reservoirs, and slows HIV-1 entry into host cells, offering potential therapeutic benefits in HIV/AIDS treatment^49^. According to our findings, porphyrins impeded the post-binding stage of virus entry and did not interfere with viral glycoprotein interaction with cellular CD4 receptors or co-receptors. Pre-incubation of the cells with compounds before the virus infection allowed them to interact with cellular receptors; however, it did not affect the virus entry. The time of addition and temperature arrest assays demonstrated that these compounds targeted the viral envelope proteins. It implied that the carboxyphenyl porphyrins may act on the post-binding or attachment events of the virus entry. Even though these derivatives exerted no effect on the HIV-1 gene expression or release, they significantly rendered the produced virus non-infectious. Several microbes and viruses have been reported to be inactivated by photodynamic therapy (PDT). PDT employs photosensitizer molecules that generate reactive oxygen species (ROS) when exposed to light and molecular oxygen. These ROS species cause oxidative stress and irreversible oxidative damage to the DNA, proteins, and lipids. Porphyrins act as photosensitizers by absorbing photons and producing ROS, providing them with bactericidal and virucidal characteristics. The advantages of the PDT like better cosmetic outcomes, non-invasiveness, minimal functional disturbances, good patient tolerance, fertility preservation, and minimization of systemic toxicity render this method more promising than classic treatment strategies like chemotherapy, radiotherapy, and surgery , which in turn, are not only energetically expensive but also pose a greater hazard to the environment^50^.

Few studies have reported photodynamic inactivation of the HIV-1 virus. **TCPP** and **THPP** have been previously reported to display antiviral activity. Under PDT conditions, carboxyphenyl porphyrins exhibited significant antiviral activity for both the HIV-1 subtypes B and C in a dose-dependent manner.

The application of PDT vastly improved the percentage of entry inhibition of the HIV-1 virus. The compounds **c-PB**_**2**_**(OH)**_**2**_ and **PB(OH)**_**3**_ at 5 µM blocked the entry of HIV-1 subtype B NL4-3 virus by more than 98% as compared to 70% in non-PDT conditions. The porphyrins **c-PB**_**2**_**(OH)**_**2**_ and **PB(OH)**_**3**_ were active against the HIV-1 subtype B NL4-3 virus during and post-virus infection under PDT conditions. In the case of HIV-1 subtype C K3016 virus, **c-PB**_**2**_**(OH)**_**2**_ and **PB(OH)**_**3**_ inhibited the virus entry by 85% when added during the infection under PDT conditions as compared to 50% in non-PDT conditions. The reference compounds **TCPP** and **THPP** have the best antiviral activity at the lower concentrations, but they are highly cytotoxic at concentrations above 3 µM under PDT conditions. On the contrary, carboxyphenyl porphyrins have significant antiviral activity and do not exhibit photocytotoxicity even at higher concentrations. Therefore, these porphyrins can be deemed more suitable for developing potent and broadly acting entry inhibitors of the HIV-1 virus.

## Conclusion

The carboxyphenyl porphyrins prevent the entry of HIV-1 subtypes B and C viruses. **c-PB**_**2**_**(OH)**_**2**_ and **PB(OH)**_**3**_ show more significant inhibitory activity than the others. These compounds' respective cis-conformation and the additional three hydroxyl groups may enhance their anti-viral potential. Under non-PDT conditions, the porphyrins act as post-attachment viral entry inhibitors. They also reduce the infectivity of the HIV-1 virus produced in their presence. The compounds exhibited better antiviral activity under the PDT conditions. At 5 µM concentration, **c-PB**_**2**_**(OH)**_**2**_ and **PB(OH)**_**3**_ restrict the virus entry by more than 98% in the case of HIV-1 subtype B and more than 85% in the case of HIV-1 subtype C virus under the PDT conditions. The compounds also inhibit the HIV-1 subtype B virus production in pre-infected TZM-bl cells exposed to photo-irradiation. In the case of HIV-1 subtype C, the virus production was reduced by 70% in the presence of porphyrins at 5 µM concentration. The carboxyphenyl porphyrins have the edge over the previously reported porphyrins **TCPP** and **THPP** due to their excellent photo-cytotoxicity values and antiviral activity under PDT conditions. Consequently, the Structure–Activity Relationship (SAR) analysis conducted in this investigation highlights the efficacy of carboxyphenyl porphyrins **c-PB**_**2**_**(OH)**_**2**_ and **PB(OH)**_**3**_ as potent inhibitors of HIV-1 entry. This underscores the importance of the proximity of two or three 4-hydroxyphenyl groups adjacent to each other at the *meso*-positions of porphyrins containing a combination of 4-hydroxyphenyl and 4-carboxyphenyl groups.

### Supplementary Information

Supplementary Information.

## Data Availability

All data generated or analyzed during this study are included in this published article (and its Supplementary Information files).
