# Author Correction: Porphyrins with combinations of 4-carboxyphenyl and 4-hydroxyphenyl substituents in *meso*-positions as anti-HIV-1 agents

**DOI:** 10.1038/s41598-024-64862-3

**Published:** 2024-06-18

**Authors:** Debdulal Sharma, Aradhana Singh, Sanaullah Safi, Ritu Gaur, Devashish Sengupta

**Affiliations:** 1 Department of Chemistry, Assam University, Silchar, Assam 788011 India; 2 Faculty of Life Sciences and Biotechnology, South Asian University, New Delhi, 110068 India

Correction to: *Scientific Reports* 10.1038/s41598-024-60728-w, published online 01 May 2024

The original version of this Article contained a display error in the Equation to determine the ${\varPhi }_{f}$ values.

$$``{\varPhi}\text{f}=\text{I}(1-10-\text{A})\text{std}{\upeta}{2}\text{Istd}(1-10-\text{A}){\upeta}\text{{std}}{2}\text{''}$$

now reads,

$${\varPhi }_{f}=\frac{I{{(1-10}^{-A})}_{std}{\eta }^{2}}{{I}_{std}{(1-10}^{-A}){\eta }_{std}^{2}}$$

The original Article has been corrected.

