# Supplementary material for: Porphyrins with combinations of 4-carboxyphenyl and 4-hydroxyphenyl substituents in meso-positions as anti-HIV-1 agents
Source: Sci Rep. 2024 May 1;14:10006. doi: 10.1038/s41598-024-60728-w (PMC11063187; doi:10.1038/s41598-024-60728-w)
Supplement: Supplementary file 1 — Supplementary Information. [file 41598_2024_60728_MOESM1_ESM.docx]

**Supporting information**

**Porphyrins with combinations of 4-carboxyphenyl and 4-hydroxyphenyl substituents in meso-positions as anti-HIV-1 agents**

Debdulal Sharma, ^#a^ Aradhana Singh,^#b^ Sanaullah Safi,^b^ Ritu Gaur^*b^ and Devashish Sengupta*^a^

^a^Department of Chemistry, Assam University, Silchar-788011, Assam, India

^b^Faculty of Life Sciences and Biotechnology, South Asian University, New Delhi-110068, India.

Corresponding Author

*Devashish Sengupta, E-mail: devashish.sengupta@uni.sydney.edu.au.

*Ritu Gaur. E-Mail: rgaur@sau.ac.in.

**1 Synthesis:**

Methyl 4-formylbenzoate, 4-hydroxybenzaldehyde, sodium hydroxide, propionic acid (Sigma-Aldrich), were used without further purification. Pyrrole (Sigma-Aldrich) was freshly distilled before use. All solvents used were purified using standard purification and drying techniques. Silica gel (60-200 mesh, & 100–200 mesh, SRL) were employed for column chromatography.

UV-visible absorption spectra were recorded using a PerkinElmer LAMBDA 750 UV/vis/NIR spectrophotometer, and emission spectra were recorded using a Fluoromax-4 spectrofluorometer (Horiba Scientific). ^1^HNMR data were recorded with a Bruker AVANCE-III 500 MHz spectrometer or a Bruker Avance III 400 MHz spectrometer or a Bruker Av III HD (DRX) 300 MHz spectrometer in CDCl_3_ or DMSO-d6 with TMS as an internal standard. MALDI-TOF spectra were recorded with a Bruker Autoflex max LRF mass spectrometer.

**1.1 Synthesis of 5,10,15-tri-(4-methoxycarbonylphenyl)-20-(4-hydroxyphenyl) porphyrin** (**PBe_3_OH**)**:**

A 500 mL amber-coloured round bottom (RB) flask fitted with a water-cooled reflux condenser attached to a drying tube was charged with propionic acid (180 mL). The contents of the flask were set to reflux for 30 minutes. After that, the system’s temperature was brought down to 70 °C followed by the addition of methyl-4-formylbenzoate (7.57 g, 46 mmol, 3.2 eq) and 4-hydroxybenzaldehyde (3.17 g, 26 mmol, 1.8 eq). Pyrrole (4 mL, 57 mmol, 4 eq) was then added drop by drop over a period of twenty minutes. The reaction set-up was set to reflux for a period of 90 minutes. On completion of the reaction, excess of propionic acid was removed by distillation. The crude product remaining in the reaction vessel was neutralised using 0.5 M ammonia and washed with deionised water, followed by warm diethyl ether. The product mixture so obtained was kept overnight and then loaded onto a silica gel column for separation. The column was initially run with 20% hexane in DCM, followed by plain DCM. The desired compound was eluted out with DCM. The product-laden fraction collected was concentrated, and a second column was run for further purification with DCM as eluent. The pure fraction collected was concentrated on a rotary evaporator, and the concentrate was resuspended in DCM. The gradual addition of hexane precipitated out the desired compound. Colour: Purple, Yield: 0.950 g, 8%.

**PBe_3_OH**: UV-Vis (DMF, 1 μM solution): 649 nm, (λ_max_; Q-band), 592 nm (λ_max_; Q-band), 551 nm (λ_max_; Q-band), 515 nm (λ_max_; Q-band), 420 nm (λ_max_; Soret band). Emission (DMF, 1 μM solution), λ_ex_= 420 nm (S-band): 645 nm, 668 nm Q(0,0), 721 nm Q(0,1) with Stokes shift 301 nm Q(0,1)-B(0,0); ^1^H-NMR (500 MHz, CDCl_3_, 298.05 K), δ: 8.94 (d, 2H, *J* = 4.8 Hz, β-Pyrrole), 8.85 (s, 4H, β-Pyrrole), 8.81 (d, 2H, *J* = 4.8 Hz, β-Pyrrole), 8.46 (d, 6H, *J* = 8 Hz), 8.31 (d, 6H, *J* = 8 Hz,), 8.07 (d, 2H, *J* = 8.3 Hz), 7.24 (d, 2H, *J* = 8.3 Hz), 4.14 (s, 9H, O-CH_3_), -2.76 (s, 2H, NH-pyrrole); ^13^C (400 MHz, CDCl_3_) δ: 167.35, 155.79, 146.84, 135.75, 134.55, 130.00, 129.89, 128.64, 127.97, 120.88, 119.71, 119.14, 113.85, 52.49. MALDI-TOF Calculated for C_50_H_36_N_4_O_7_ [M+H]^+^ m/z 805.2662, found 805.038.

The one-pot synthesis also led to the formation of 5,10-di-(4-methoxycarbonylphenyl)-15,20-di-(4-hydroxyphenyl) porphyrin (**c-PBe_2_(OH)_2_**), 5,15-di-(4-methoxycarbonylphenyl)-10,20-di-(4-hydroxyphenyl) porphyrin (**t-PBe_2_(OH)_2_**) and 5-tri-(4-methoxycarbonylphenyl)-10,15,20-tri-(4-hydroxyphenyl) porphyrin (**PBe(OH)_3_**), all of which were isolated, purified and characterised.

**c-PBe_2_(OH)_2_:** UV-Vis (DMF, 10 μM solution): 650 nm, (λ_max_; Q-band), 594 nm (λ_max_; Q-band), 554 nm (λ_max_; Q-band), 517 nm (λ_max_; Q-band), 422 nm (λ_max_; Soret band). Emission (DMF, 10 μM solution), λ_ex_= 422 nm (S-band): 658 nm Q(0,0), 722 nm Q(0,1) with Stokes shift 236 nm Q(0,0)-B(0,0); ^1^H-NMR (500 MHz, CDCl_3_, 298.05 K), δ: 8.92 (d, 2H, *J* = 4.5 Hz, β-pyrrole), 8.9 (s, 2H), 8.81 (s, 2H), 8.8 (d, 2H, *J* = 4.5 Hz, β-pyrrole), 8.46 (d, 4H, *J* = 8 Hz), 8.32 (d, 4H, *J* = 8 Hz,), 8.08 (d, 4H, *J* = 8.3 Hz), 7.24 (d, 4H, *J* = 8.3 Hz), 4.14 (s, 6H), −2.76 (s, 2H, NH-pyrrole); δ: MALDI-TOF Calculated for C_48_H_34_N_4_O_7_ [M] m/z 762.2478, found 762.291.

**t-PBe_2_(OH)_2_:** UV-Vis (DMF, 10 μM solution): 649 nm, (λ_max_; Q-band), 594 nm (λ_max_; Q-band), 552 nm (λ_max_; Q-band), 515 nm (λ_max_; Q-band), 422 nm (λ_max_; Soret band). Emission (DMF, 10 μM solution), λ_ex_= 422 nm (S-band): 660 nm Q(0,0), 722 nm Q(0,1) with Stokes shift 238 nm Q(0,0)-B(0,0); ^1^H-NMR (400 MHz, CDCl_3_, 298.05 K), δ: 8.90 (d, 4H, *J* = 4.6 Hz, β-Pyrrole), 8.78 (d, 4H, *J* = 4.6 Hz, β-Pyrrole), 8.44 (d, 4H, *J* = 7.8 Hz), 8.30 (d, 4H, *J* = 7.8 Hz), 8.06 (d, 4H, *J* = 8.1 Hz,), 7.23 (d, 4H, *J* = 8.1 Hz), 4.12 (s, 6H), 3.89 (s, br, 2H, -OH), -2.77 (s, 2H, NH-pyrrole); δ: MALDI-TOF Calculated for C_48_H_34_N_4_O_7_ [M] m/z 762.2478, found 762.874

**PBe(OH)_3_:** UV-Vis (DMF, 10 μM solution): 649 nm, (λ_max_; Q-band), 602 nm (λ_max_; Q-band), 560 nm (λ_max_; Q-band), 519 nm (λ_max_; Q-band), 428 nm (λ_max_; Soret band). Emission (DMF, 10 μM solution), λ_ex_= 428 nm (S-band): 616, 660 nm Q(0,0), 721 nm Q(0,1) with Stokes shift 232 nm Q(0,0)-B(0,0); ^1^H-NMR (400 MHz, CDCl_3_, 298.05 K), δ: 8.91 (d, 2H, *J* = 4.9 Hz, β-Pyrrole), 8.90 (s, 4H, β-Pyrrole), 8.79 (d, 2H, *J* = 4.9 Hz, β-Pyrrole), 8.46 (d, 2H, *J* = 8.1 Hz), 8.32 (d, 2H, *J* = 8.1 Hz), 8.09 (d, 6H, *J* = 8.3 Hz), 7.24 (d, 6H, *J* = 8.3 Hz), 4.14 (s, 3H, O-CH_3_), -2.74 (s, 2H, NH-pyrrole); δ: MALDI-TOF Calculated for C_46_H_32_N_4_O_5_ [M+H]^+^ m/z 721.7928, found 721.346.

**1.2 Synthesis of 5,10,15-tri-(carboxyphenyl)-20-(4-hydroxyphenyl) porphyrin (PB_3_OH):**

Compound **PBe_3_OH** (0.084 g, 0.104 mmol, 1 eq) was dissolved in DMF (21.8 mL) in a 150 mL single neck round bottom flask. The flask was then charged with crushed NaOH (0.87 g, 21.75 mmol, 209 eq) and the contents were subjected to stirring for 30 min. Thereafter deionized water (80 mL) was added and stirring continued for 30 more minutes. The pH of the reaction mixture was then brought down to 4.5, using 6 N HCl, when the product finally precipitated out. The precipitate was recovered through centrifugation, washed repeatedly with MeOH, water and dried. Yield: 0.068 g,. 85%.

**PB_3_OH:** UV-Vis (Water, 10 μM solution): 653 nm (λ_max_; Q-band), 598 nm (λ_max_; Q-band), 559 nm (λ_max_; Q-band), 523 nm (λ_max_; Q-band), 421 nm (λ_max_; Soret band). Emission (Water, 10 μM solution), λ_ex_= 421 nm (S-band): 614 nm, 662 nm Q(0,0) with Stokes shift 241 nm ($\Delta\lambda= \lambda_{\mathrm{em}}^{\max}- \lambda_{\mathrm{abs}}^{\max}$); ^1^H-NMR (300 MHz, DMSO-d6, 300 K), δ: 8.92 (d, 2H, *J* = 5 Hz, β-Pyrrole), 8.82 (s, 4H, β-Pyrrole), 8.77 (d, 2H, *J* = 5 Hz, β-Pyrrole) 8.36 (d, 6H, *J* = 8.3 Hz), 8.28 (d, 6H, *J* = 8.3 Hz) 8.00 (d, 2H, *J* = 8.5 Hz), 7.22 (d, 2H, *J* = 8.5 Hz), -2.90 (s, 2H, NH-pyrrole); MALDI-TOF Calculated for C_47_H_30_N_4_O_7_ [M+2H]^+^ m/z 764.227, found 764.089.

A similar procedure was employed to obtain 5,10-di-(4-carboxyphenyl)-15,20-di-(4-hydroxyphenyl) porphyrin (**c-PB_2_(OH)_2_)**, 5,15-di-(4-carboxyphenyl)-10,20-di-(4-hydroxyphenyl) porphyrin (**t-PB_2_(OH)_2_**), and 5,10,15-di-(4-carboxyphenyl)-20-(4-hydroxyphenyl) porphyrin **PB(OH)_3_,** the hydrolysis product of the free-base esters **c-PBe_2_(OH)_2_**, **t-PBe_2_(OH)_2_**, and **PBe(OH)_3_**, respectively.

**c-PB_2_(OH)_2_:** UV-Vis (Water, 10 μM solution): 690, (λ_max_; Q-band)~~,~~ 600, (λ_max_; Q-band), 562 (λ_max_; Q-band), 524 (λ_max_; Q-band), 419 (λ_max_; Soret band). Emission (Water, 10 μM solution), λ_ex_= 419 nm (S-band): 612, 654 (0,0), 710 (0,1) with Stokes shift 235 nm Q(0,0)-B(0,0); ^1^H-NMR (400 MHz, DMSO-d6, 298.05 K), δ: 8.83-8.92 (m, 8H, β-Pyrrole) 8.38 (d, 4H, *J* = 7.3 Hz), 8.31 (d, 4H, *J* = 7.3 Hz) 8.02 (d, 4H, *J* = 7.1 Hz), 7.23 (d, 4H, *J* = 7.1 Hz), -2.87 (s, 2H, NH-pyrrole); Calculated for C_46_H_30_N_4_O_6_ [M+H]^+^ m/z 734.2165, found 734.855

**t-PB_2_(OH)_2_:** UV-Vis (DMF, 10 μM solution): 662, (λ_max_; Q-band)~~,~~ 601, (λ_max_; Q-band), 560 (λ_max_; Q-band), 522 (λ_max_; Q-band), 426 (λ_max_; Soret band). Emission (DMF, 10 μM solution), λ_ex_= 426 nm (S-band): 612, 653 (0,0), 708 (0,1) with Stokes shift 228 nm Q(0,0)-B(0,0); ^1^H-NMR (400 MHz, DMSO-d6, 298.05 K), δ: 12.81 (s, 2H), 8.91 (d, 4H, *J* = 5.1 Hz, β-Pyrrole), 8.81 (d, 4H, *J* = 5.6 Hz, β-Pyrrole), 8.38 (d, 4H, *J* = 8 Hz), 8.33 (d, 4H, *J* = 8 Hz), 7.21 (d, 4H, *J* = 7.6 Hz), 6.90 (d, 4H, *J* = 8 Hz), 3.58 (s, br, 2H), −2.91 (s, 2H, NH-pyrrole); Calculated for C_46_H_30_N_4_O_6_ [M+H]^+^ m/z 734.2165, found 734.554

**PB(OH)_3_:** UV-Vis (Water, 10 μM solution): 684, (λ_max_; Q-band)~~,~~ 602, (λ_max_; Q-band), 567 (λ_max_; Q-band), 525 (λ_max_; Q-band), 420 (λ_max_; Soret band). Emission (Water, 10 μM solution), λ_ex_= 420 nm (S-band): 614, 657 (0,0), 710 (0,1) with Stokes shift 237 nm Q(0,0)-B(0,0); ^1^H-NMR (400 MHz, DMSO-d6, 298.05 K), δ: 9.95 (d, 2H, *J* = 6.1 Hz, β-Pyrrole), 8.82 (s, 4H, β-Pyrrole), 8.79 (d, 2H, *J* = 2.4 Hz, β-Pyrrole), 8.38 (d, 6H, *J* = 7.6 Hz), 8.33 (d, 6H, *J* = 7.6 Hz), 8.00 (d, 2H, *J* = 7.3 Hz), 7.20 (d, 2H, *J* = 7.3 Hz), -2.88 (s, 2H, NH-pyrrole). MALDI-TOF: Calculated for C_46_H_30_N_4_O_6_ [M+H]^+^ m/z 706.7580, found 706.863

**2. Methods:**

**2.1. Cell lines and plasmids**: HEK-293T and TZM-bl cells were cultured at 37 °C with 5% CO_2_ in Dulbecco's modified Eagle's media supplemented with 10% FBS and 1% penicillin, streptomycin antibiotic solution. The TZM-bl cell line is a derivative of the HeLa cell line, and it is modified to express CD4, CCR5, and CXCR4. This cell line is very susceptible to infection by most HIV, SIV, and SHIV strains. It has separate copies of the luciferase and -galactosidase genes controlled by the HIV-1 long terminal repeat promoter. HIV infection may be precisely measured as a function of luciferase or β-galactosidase activity.^1-2^ The TZM-bl cell lines, HIV-1 subtype B molecular clones NL4-3, and NL4-3 env- generously provided by Dr. Eric O. Freed (National Cancer Institute, NIH, USA). The HIV-1 subtype C clone, K3016, was a generous gift from Dr. Christina Ochsenbauer, University of Alabama, USA; Gene Bank accession: KC156129).

**2.2. Preparation of test compounds:** All the compounds were prepared in 5% DMSO in water (-) solution. T20 (Enfuvirtide) stock solution was prepared by using only DMSO. The antiviral activity of the compounds was investigated in dark non-photodynamic (non-PDT) and photodynamic (PDT ) conditions under specific conditions.

**2.3. Cytotoxicity Assay**: To analyze the effect of these compounds on cell viability, the cytotoxicity of the compounds was tested against TZM-Bl cells and HEK293T cells. The cytotoxicity test was carried out according to the manufacturer's instructions using the Cell Titre-Blue Cell Viability Assay kit (Promega, USA). 7000 cells/well were cultured on an opaque white 96-well plate with serial dilutions of compounds for 48 h in the dark before being treated with 20 µl/well Cell Titre-Blue reagent for 4 h at 37 °C. The concentrations employed for toxicity tests on HEK293T and TZM-bl cells varied from 0.1 µM to 50 µM. A Bio Tek microplate reader was used to record the fluorescent signals at 530/25 excitation and 590/35 emission. For testing the effect of compounds on the cell viability under photodynamic (PDT) conditions, HEK-293T and TZM-bl cells were incubated with compounds at concentrations ranging from 0.1 µM to 50 µM, followed by 45 minutes of light irradiation at a distance of 6 inches inside an experimentally designed light chamber. A light-only control with untreated cells was incorporated to check the effect of light irradiation on the untreated cells. After 48 h of incubation at 37 °C, the viability of the treated cells was compared to that of the untreated cells.

**2.4. Viral gene expression studies:** HEK-293T cells were cultured to about 50%-60% confluency in a 6-well culture plate, then transfected with 3 μg of HIV-1 Subtype B NL4-3 or subtype C K3016DNA and generated in the presence or absence of 500nM to 5 μM compounds. The viral supernatant was collected 24 h after transfection, centrifuged, and filtered to remove residual cell debris. The cells that had been transfected were washed and collected in 1X phosphate-buffered saline (PBS). After centrifugation at 845 g for 5 minutes, the cell pellets were lyzed in 1X RIPA (radioimmunoprecipitation assay) buffer. 50mM Tris-HCl pH 8.0, 150mM sodium chloride, 1.0% NP-40, 0.5% sodium deoxycholate, 0.1% SDS, and 1X protease inhibitor cocktail (Roche, Germany) are used to make 1X RIPA buffer.

**2.5. Virus infectivity assay:** A TZM-bl cell-based single-cycle infectivity assay was used to assess the infectivity of the viruses produced in the presence or absence of porphyrins.^62,63^.TZM-bl cells were infected with 10ng of HIV-1 subtype B NL4-3 or subtype C K3016 p24 equivalent virus for 2 h. After infection, the cells were washed and incubated for 48 h without test compounds at 37 °C. Relative luminescence was measured 48 h post-infection and compared to the control. The infectivity of HIV-1 subtype B NL4-3 virus or HIV-1 subtype C K3016 virus produced in the presence of 5% DMSO in water (-), in the absence of the carboxyphenyl porphyrins, served as the treatment control.

**2.6. Entry inhibition assay in TZM-bl cells under non-PDT conditions:** NL4-3/K3016 viral stocks were generated in HEK293T cells. After 24 h, the viral supernatant was collected, centrifuged, and filtered to remove any remaining cell debris. The HIV-1 p24 Antigen Capture Kit was used to quantify the virus. The p24 normalized virus was utilized to infect TZM-bl cells in the presence of 20 µg DEAE-dextran per ml (7X10^4^/well). The concentration of the compounds was used in the range of 100 nM to 5µM. Three distinct conditions were used for the infection: A) Compounds were added to the cells during virus infection. B) The compounds were introduced to the cells after virus infection and incubated for 48 h.T-20, an HIV-1 fusion inhibitor, was utilized as a positive control. C). TZM-bl cells were pre-treated with 5 µM of carboxyphenyl porphyrins for 2 h before virus infection.

The luciferase activity in cell lysates was evaluated 48 h after infection using the Steady-Glo luciferase assay kit (Promega, USA) according to the manufacturer's instructions. The percentage relative entry was obtained by normalizing the luciferase units compared to the 5% DMSO in water (-) control.

**2.7. Entry inhibition assay in T cells under non-PDT conditions:** Human T-cell line- HuTR5 cells were infected with the HIV-1 Subtype B NL4-3 or subtype C K3016virus. The infected cells were washed and cultured for 4 days for NL4-3 and 7 days for K3016 at 37 ° C in the presence or absence of compounds. The virus was then quantified using p24ELISA.

**2.8 Temperature arrest assay under non-PDT conditions:** TZM-bl cells were maintained with the compounds for 2 h at 4 °C during virus infection using spinoculation infection. The cells were subsequently washed and separated into two sets: one set was incubated with the compounds, and the other was incubated without the compounds for 48 h at 37 °C. Following the manufacturer's instructions, the relative luciferase activity was determined using the Steady-Glo luciferase assay kit (Promega, USA).

**2.9. Time of addition assay:** TZM-bl cells were infected with 10ng of HIV-1 subtype B NL4-3 p24 equivalent virus. To assess the antiviral activity of the compounds upon virus entry, compounds were introduced at -0mins, 30 mins, 1 h, and 2 h following virus addition to the cells and maintained through the course of infection. After 2 h, cells were washed and incubated for 48 h. The luciferase activity in cell lysates was evaluated 48 h after infection using the Steady-Glo luciferase assay kit (Promega, USA) according to the manufacturer's instructions. The percentage relative entry was obtained by normalizing the luciferase units compared to the 5% DMSO in water (-) control.

**2.10. Entry inhibition assay in TZM-bl cells under PDT conditions:** An irradiation box was conceptualized and designed for the PDT experiments. It had the dimensions of length 20", width 6", and height 8", and it was equipped with two Philips Essential Master PL-L 36W/865/4P linear, compact fluorescent lamps. The surface of a 96-well microplate was deemed to be 15 cm from the light source. The light emitted by the source was that of the visible light spectrum with the wavelength ranging from 400 nm to 700nm.The duration of exposure to the irradiation was for 45 minutes. 250 Jcm^-2^ of light was determined to be present at the surface. 50 ng of HIV-1 p24 equivalent HIV-1 Subtype B NL4-3 or subtype C K3016 virus was pre-treated with 10 nM to 500 nM compounds and exposed to a specific light source at a distance of 6 inches inside an experimentally designed light chamber for 45 minutes. After being pre-treated, the virus infected TZM-bl cells for 2 h at 37 °C. After infection, cells were washed and maintained for 48 h without compounds. HIV-1 fusion inhibitor T-20 was used as a positive control. A light only control with TZM-bl cells without virus and compounds was incorporated to check the effect of light irradiation on the untreated cells. The relative luminescence corresponding to HIV infection was measured. For checking the effect of porphyrins on virus production, TZM-bl cells were infected with 50ng of HIV-1 p24 equivalent HIV-1 Subtype B NL4-3 or subtype C K3016virus for 2 h at 37 °C. Following infection, cells were treated with the compounds and irradiated for 45 minutes from a distance of 6 inches inside the light chamber by a specific light source. After 48 h of incubation at 37 °C, the relative luminescence corresponding to HIV production inside the cells was measured.


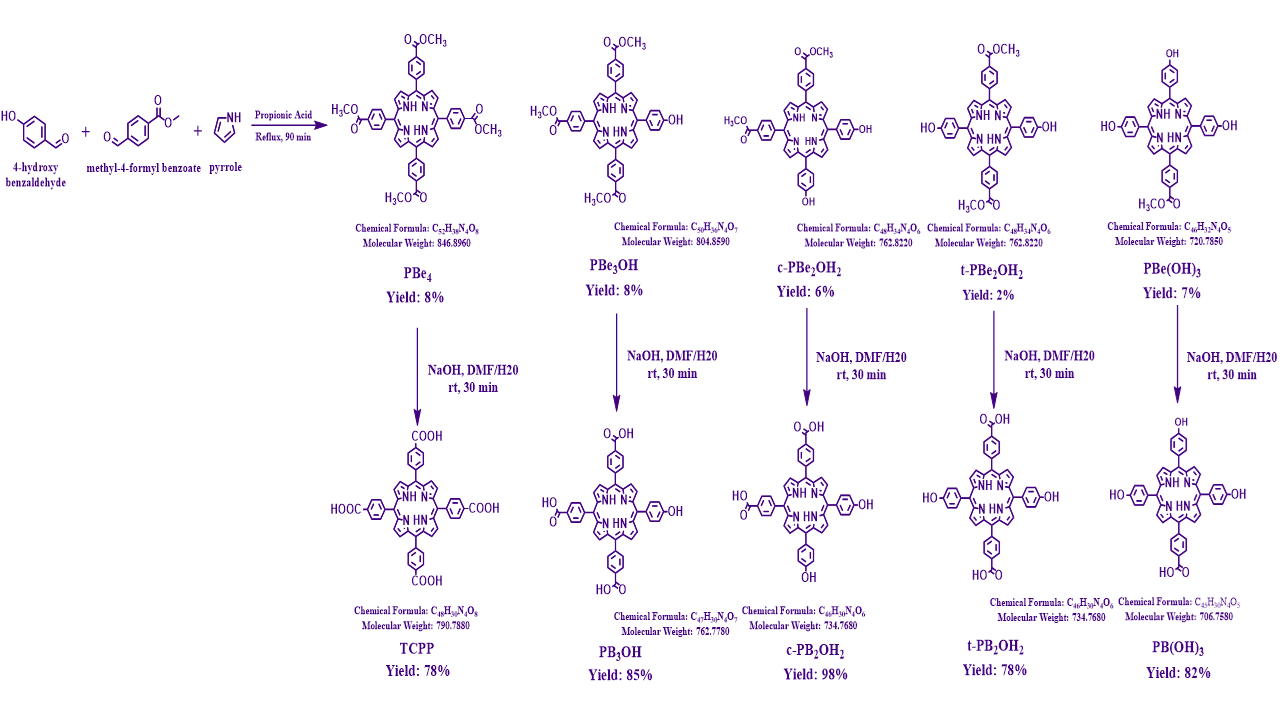


**Figure S1:** Synthetic methodology involved in the synthesis of the hydrophilic free base carboxyphenyl porphyrin derivatives **TCPP**, **PB_3_OH**, **c-PB_2_(OH)_2_**, **t-PB_2_(OH)_2_** and **PB(OH)_3_**

**Table S1:** ^1^H Chemical shift data (δ, ppm) for **PBe_3_OH**, **c-PBe_2_(OH)_2_**, **t-PBe_2_(OH)_2_** and **PBe(OH)_3_** in CDCl_3_. Multiplicity, integration and coupling constants in parentheses.

| **Code** | **H-β-pyrrolic** | **ArH** | **-OCH3** | **-NH pyrrole** |
| --- | --- | --- | --- | --- |
| **PBe_3_OH** | 8.94 (d, 2H, *J* = 4.8 Hz), 8.85 (s, 4H), 8.81 (d, 2H, *J* = 4.8 Hz), | 8.46 (d, 6H, *J* = 8 Hz), 8.31 (d, 6H, *J* = 8 Hz,), 8.07 (d, 2H, *J* = 8.3 Hz), 7.24 (d, 2H, *J* = 8.3 Hz), | 4.14 (s, 9H) | –2.76 (s, 2H) |
| **c-PBe_2_(OH)_2_** | 8.92 (d, 2H, *J* = 4.5 Hz), 8.9 (s, 2H), 8.81 (S, 2H), 8.8 (d, 2H, *J* = 4.5 Hz) | 8.46 (d, 4H, *J* = 8 Hz), 8.32 (d, 4H, *J* = 8 Hz,), 8.08 (d, 4H, *J* = 8.3 Hz), 7.24 (d, 4H, *J* = 8.3 Hz) | 4.14 (s, 6H) | −2.76 (s, 2H, pyrrole) |
| **t-PBe_2_(OH)_2_** | 8.90 (d, 4H, *J* = 4.6 Hz), 8.78 (d, 4H, *J* = 4.6 Hz) | 8.44 (d, 4H, *J* = 7.8 Hz), 8.30 (d, 4H, *J* = 7.8 Hz), 8.06 (d, 4H, *J* = 8.1 Hz,), 7.23 (d, 4H, *J* = 8.1 Hz) | 4.12 (s, 6H) | –2.76 (s, 2H) |
| **PBe(OH)_3_** | 8.91 (d, 2H, *J* = 4.9 Hz), 8.90 (s, 4H, β-Pyrrole), 8.79 (d, 2H, *J* = 4.9 Hz) | 8.46 (d, 2H, *J* = 8.1 Hz), 8.32 (d, 2H, *J* = 8.1 Hz), 8.09 (d, 6H, *J* = 8.3 Hz), 7.24 (d, 6H, *J* = 8.3 Hz) | 4.14 (s, 3H) | –2.74 (s, 2H) |

**Table S2:** UV-Vis and Emission data of **PBe_3_OH**, **c-PBe_2_(OH)_2_**, **t-PBe_2_(OH)_2_** and **PBe(OH)_3_**.

| **Code** | **Solvent/**  **Concentration** | **Absorption (nm)** | **Emission** | |
| --- | --- | --- | --- | --- |
|  |  | **λ_abs_/nm (ε/10^3^ M^−1^ cm^−1^)** | **λ_em/_**  **nm** | **Stokes shift** |
| **PBe_3_OH** | DMF/1 μM | 420 (394.3), 515 (17.3), 551 (9.6), 592 (5.4), 649 (6.2) | 645, 668, 721 | 301 |
| **c-PBe_2_(OH)_2_** | DMF/10 μM | 422 (316.8), 517 (23.7), 554 (16.9), 594 (8.6), 650 () | 658, 722 | 236 |
| **t-PBe_2_(OH)_2_** | DMF/10 μM | 422 (10.7), 515 (1.4), 552 (1.1), 594 (0.8), 649 (0.7) | 660, 722 | 238 |
| **PBe(OH)_3_** | DMF/10 μM | 428 (315.7), 519 (7.9), 560 (21.5), 602 (13.2), 649 (2.4). | 616, 660, 721 | 232 |

**Figure S2:** UV-Vis and emission spectral plot of **PBe_4_, PBe_3_OH**, **c-PBe_2_(OH)_2_**, **t-PBe_2_(OH)_2_** and **PBe(OH)_3_** recorded in DMF at a concentration of 10 μM.

**Table S3:** ^1^H Chemical shift data (δ, ppm) for **PB_3_OH**, **c-PB_2_(OH)_2_**, **t-PB_2_(OH)_2_** and **PBe(OH)_3_** in DMSO-d6. Multiplicity, integration and coupling constants in parentheses.

| **Code** | **−COOH** | **H-β-pyrrolic** | **ArH** | **−OH** | **−NH** |
| --- | --- | --- | --- | --- | --- |
| **PB_3_OH** |  | 8.92 (d, 2H, *J* = 5 Hz), 8.82 (s, 4H), 8.77 (d, 2H, *J* = 5 Hz,) | 8.36 (d, 6H, *J* = 8.3 Hz), 8.28 (d, 6H, *J* = 8.3 Hz) 8.00 (d, 2H, *J* = 8.5 Hz), 7.22 (d, 2H, *J* = 8.5 Hz) |  | –2.90 (s, 2H, NH-pyrrole) |
| **c-PB_2_(OH)_2_** | − | 8.83-8.92 (m, 8H) | 8.38 (d, 4H, *J* = 7.3 Hz), 8.31 (d, 4H, *J* = 7.3 Hz) 8.02 (d, 4H, *J* = 7.1 Hz), 7.23 (d, 4H, *J* = 7.1 Hz) | − | –2.87 (s, 2H, NH-pyrrole) |
| **t-PB_2_(OH)_2_** | 12.81 | 8.91 (d, 4H, *J* = 5.1 Hz), 8.81 (d, 4H, J = 5.6 Hz). | 8.38 (d, 4H, *J* = 8 Hz), 8.33 (d, 4H, *J* = 8 Hz), 7.21 (d, 4H, *J* = 7.6 Hz), 6.90 (d, 4H, *J* = 8 Hz). | 3.58  (s, br, 2H) | –2.91 (s, 2H, NH-pyrrole) |
| **PB(OH)_3_** | − | 9.95 (d, 2H, *J* = 6.1 Hz), 8.82 (s, 4H), 8.79 (d, 2H, *J* = 2.4 Hz,) | 8.38 (d, 6H, *J* = 7.6 Hz), 8.33 (d, 6H, *J* = 7.6 Hz) 8.00 (d, 2H, *J* = 7.3 Hz), 7.20 (d, 2H, *J* = 7.3 Hz) | − | –2.88 (s, 2H, NH-pyrrole) |


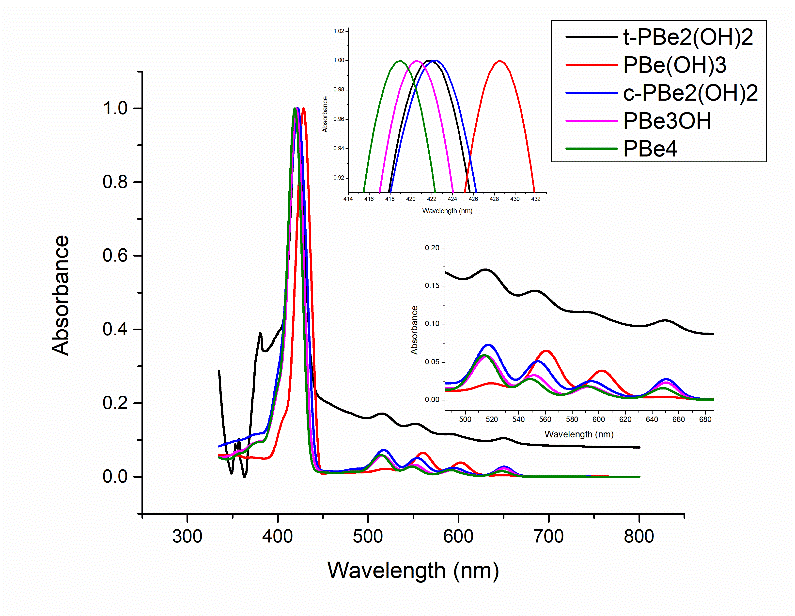

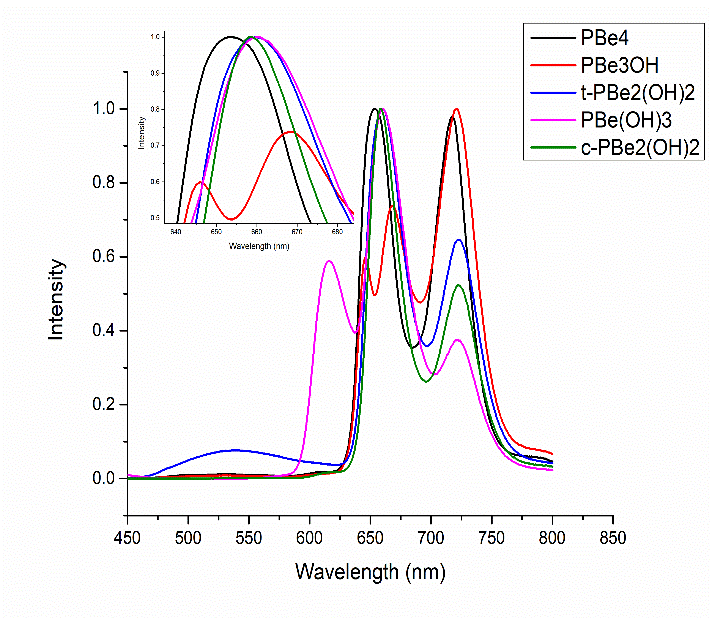


**A**

**B**

**Figure S3:** UV-Vis and emission spectral plot of **c-PB_2_(OH)_2_, t-PB_2_(OH)_2_, PB(OH)_3_**, **PB_3_OH** and **TCPP** recorded in water at a concentration of 10 μM.

**Table S4:** UV and emission spectral data of **PB_3_OH**, **c-PB_2_(OH)_2_**, **t-PB_2_(OH)_2_**, **PB(OH)_3_**, and **TCPP** recorded in water at a concentration of 10 μM.


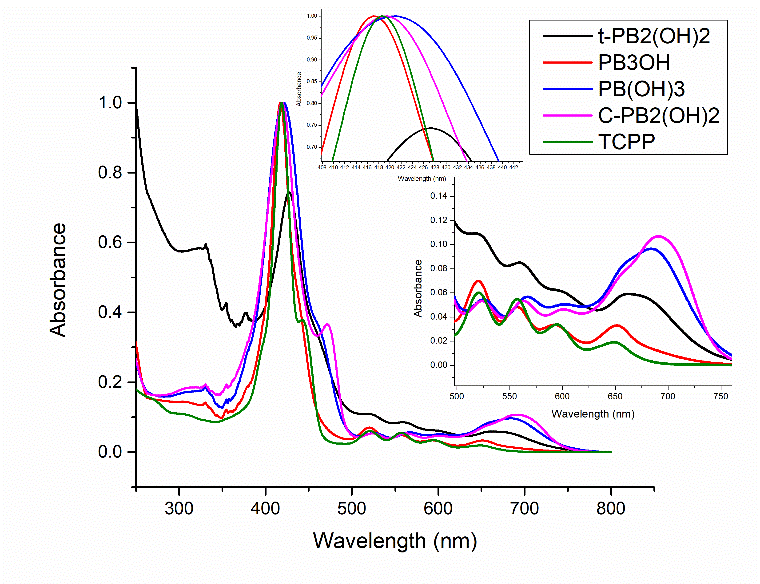

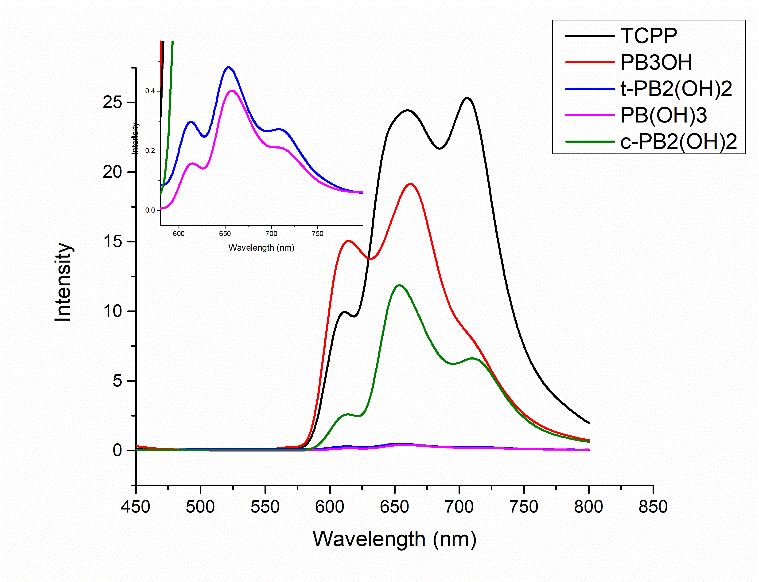


**A**

**B**

| **Code** | **Solvent/**  **Concentration** | **Absorption (nm)** | **Emission** | |
| --- | --- | --- | --- | --- |
|  |  | **λ_abs_/nm (ε/10^3^ M^−1^ cm^−1^)**  B(0,0), Qy(1,0), Qy(0,0), Qx(1,0), Qx(0,0) | **λ_em_/nm**  -, Q(0,0), Q(0,1) | **Stokes shift***  **(nm)** |
| **PB_3_OH** | Water/10 μM | 421 (112.6), 523 (9.1), 559 (10.8), 598 (7.9), 653 (5.4) | 614, 662, -, | 241 |
| **c-PB_2_(OH))_2_** | Water/10 μM | 419 (65.0), 524 (3.7), 562 (3.7), 600 (3.2), 690 (7.2) | 612, 654, 710 | 235 |
| **t-PB_2_(OH))_2_** | Water/10 μM | 426 (20.0), 522 (3.2), 560 (2.6), 601 (1.9), 662 (1.9). | 612, 654, 708 | 228 |
| **PB(OH)_3_** | Water/10 μM | 420 (48.7), 525 (2.8), 567 (2.9), 602 (2.7), 684 (4.9) | 614, 657, 710 | 237 |
| **TCPP** | Water/10 μM | 418 (195.7), 520 (12.5), 557 (11.4), 594 (7.3), 649 (4.4). | 611, 660, 706 | 288 |

*Stokes shifts were calculated as $\Delta\lambda= \lambda_{\mathrm{em}}^{\max}- \lambda_{\mathrm{abs}}^{\max}$^3-4^

**2 Characterization:**

**2.1 ^1^H-NMR Spectra:**


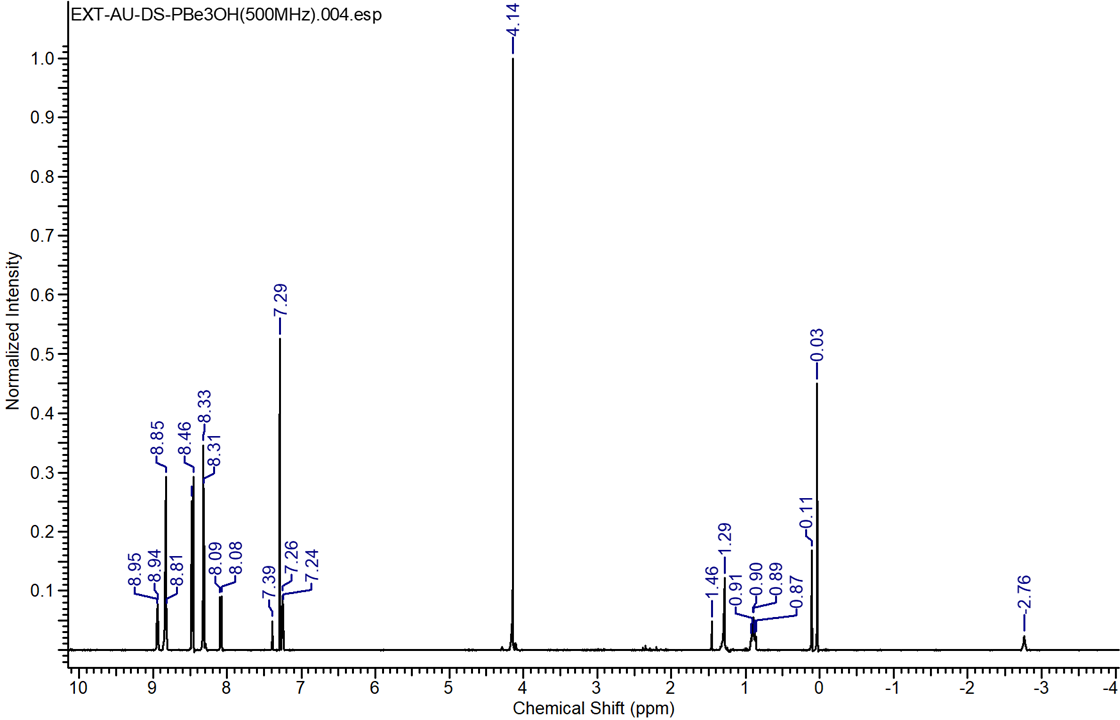

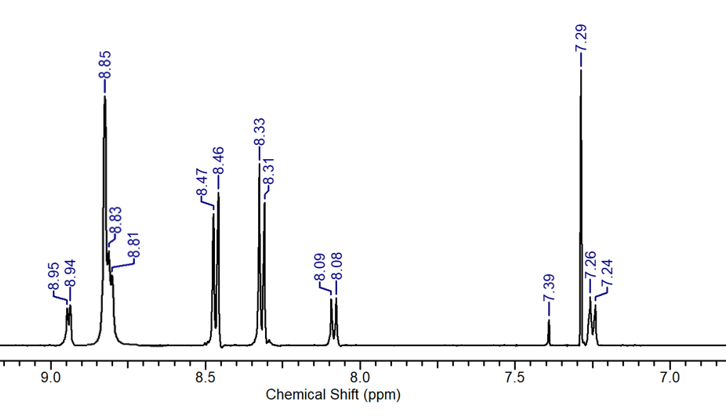


**Figure S4:** ^1^H-NMR Spectra: 5,10,15-tri-(4-methoxycarbonylphenyl)-20-(4-hydroxyphenyl) porphyrin (**PBe_3_OH**)

**
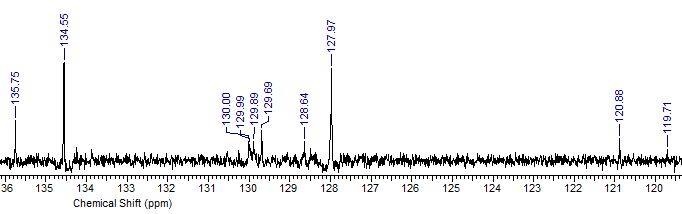
Figure S5:** ^13^C-NMR Spectra: 5,10,15-tri-(4-methoxycarbonylphenyl)-20-(4-hydroxyphenyl) porphyrin (**PBe_3_OH**)

**Figure S6:** ^1^H-NMR Spectra: 5,10-tri-(4-methoxycarbonylphenyl)-15,20-(4-hydroxyphenyl) porphyrin (**c-PBe_2_(OH)_2_**)

**Figure S7:** ^1^H-NMR Spectra: 5,15-tri-(4-methoxycarbonylphenyl)-10,20-(4-hydroxyphenyl) porphyrin (**t-PBe_2_(OH)_2_**)

**Figure S8:** ^1^H-NMR Spectra: 5-(4-methoxycarbonylphenyl)-10,15,20-(4-hydroxyphenyl) porphyrin (**PBe(OH)_3_**)

**Figure S9:** ^1^H-NMR Spectra: 5,10,15-tri-(4-carboxyphenyl)-20-(4-hydroxyphenyl) porphyrin (**PB_3_OH**)


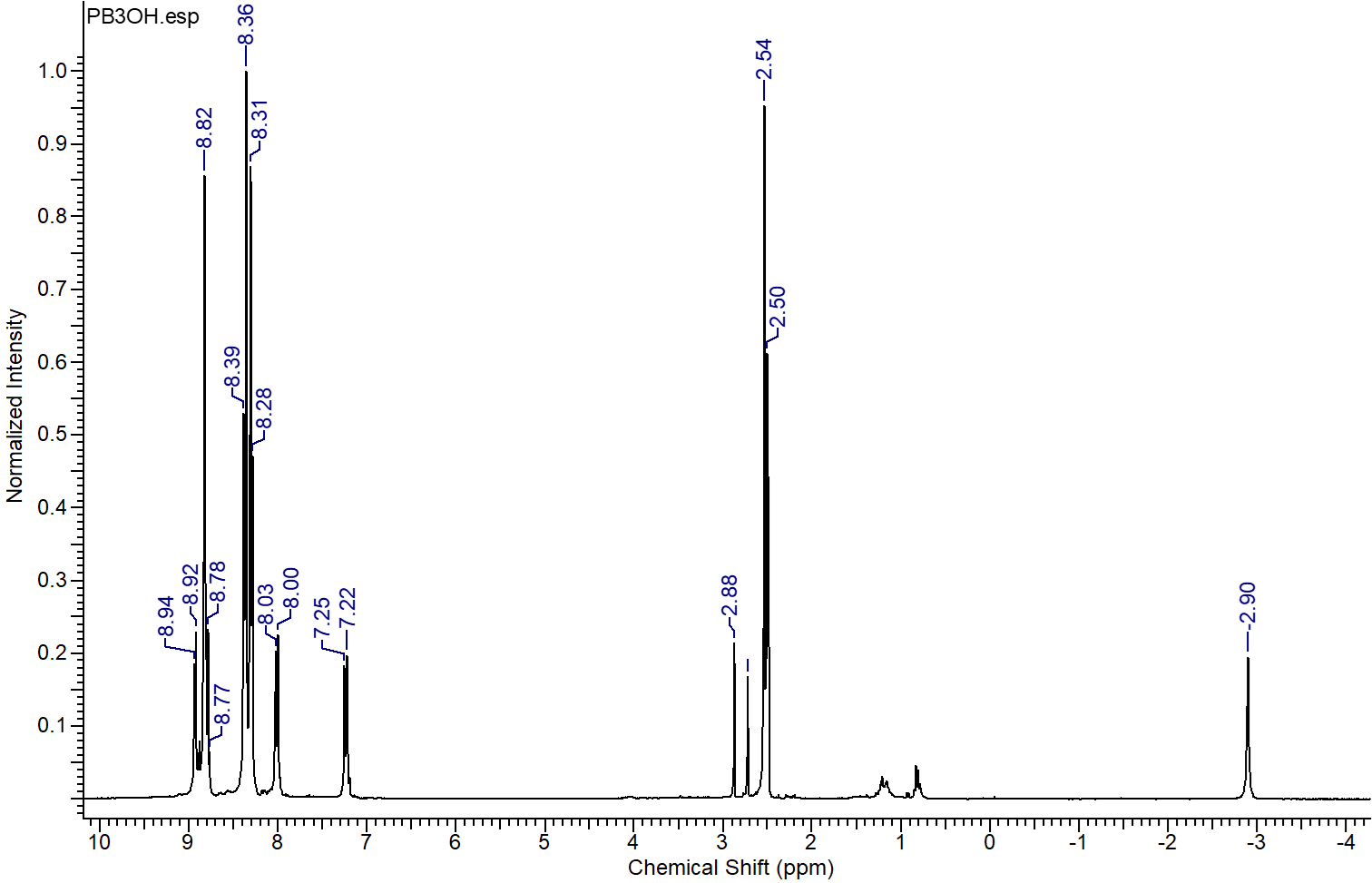

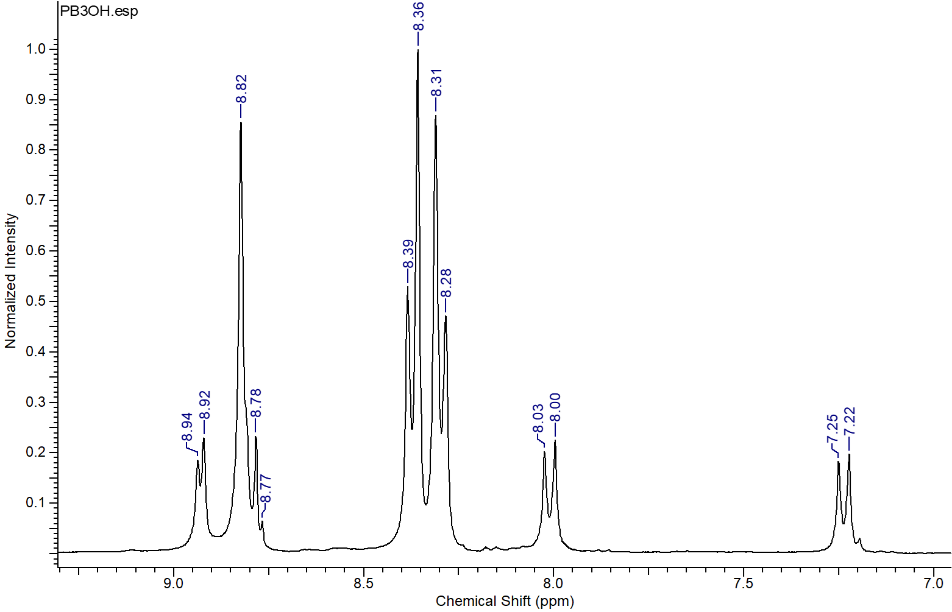

**Figure S10:** ^1^H-NMR Spectra: 5,10-di-(4-carboxyphenyl)-15,20-di-(4-hydroxyphenyl) porphyrin (**c-PB_2_(OH)_2_**)

**
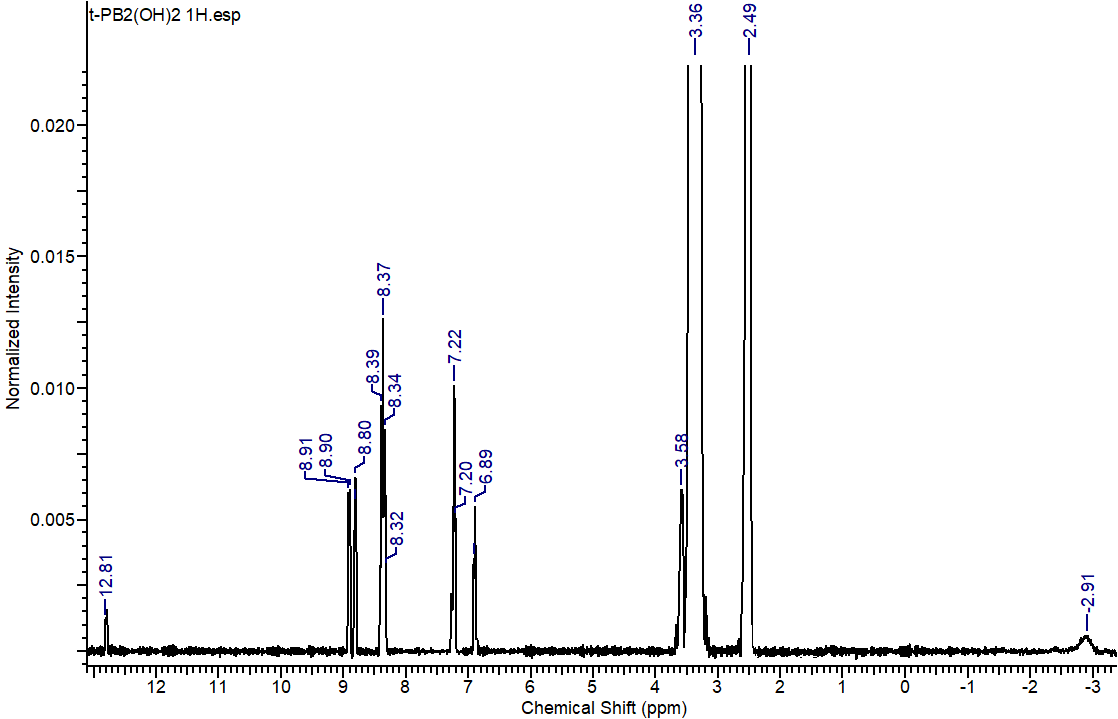
**

**
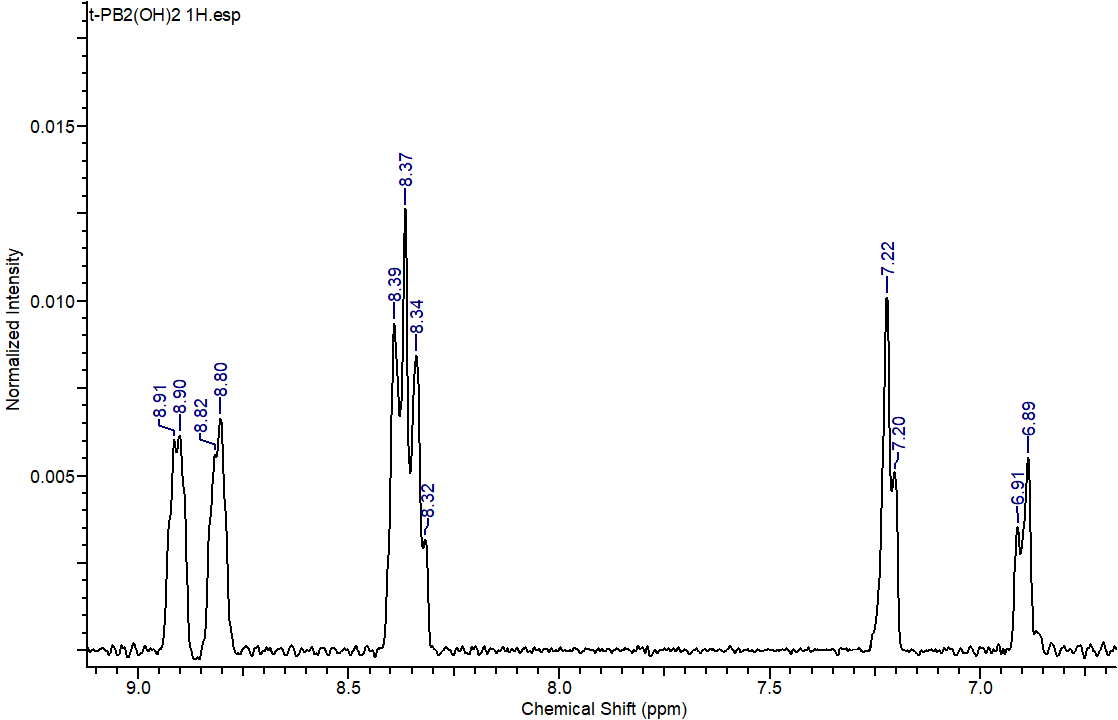
**

**Figure S11:** ^1^H-NMR Spectra: 5,15-di-(4-carboxyphenyl)-10,20-di-(4-hydroxyphenyl) porphyrin (**t-PB_2_(OH)_2_**)

**Figure S12:** ^1^H-NMR Spectra: 5-(4-carboxyphenyl)-10,15,20-tri-(4-hydroxyphenyl) porphyrin (**PB(OH)_3_**)

**2.2 Mass Spectra:**


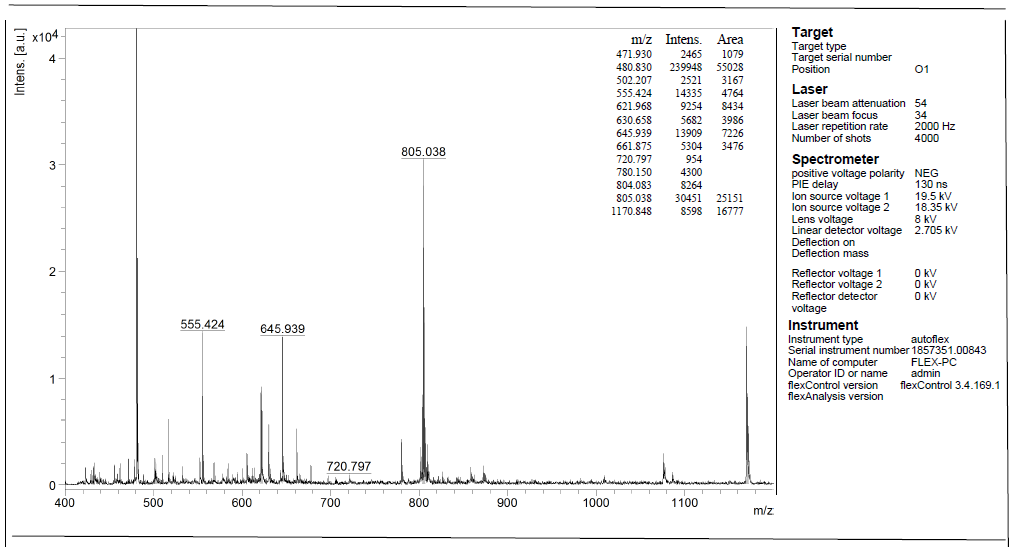


**
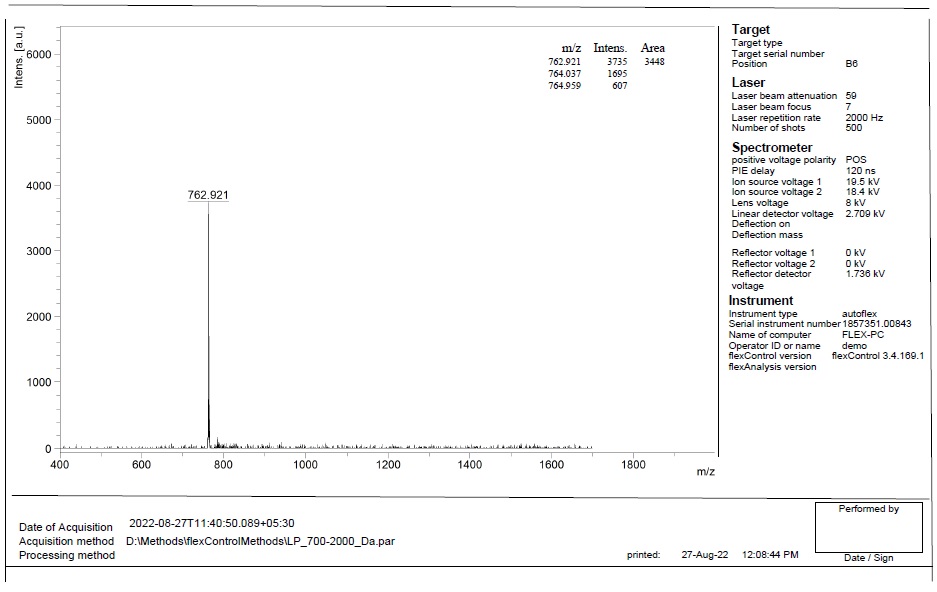
Figure S13:** MALDI-TOF Spectra: 5,10,15-tri-(4-methoxycarbonylphenyl)-20-(4-hydroxyphenyl) porphyrin (**PBe_3_OH**)

**Figure S14:** MALDI-TOF Spectra: 5,10-di-(4-methoxycarbonylphenyl)-15,20-di-(4-hydroxyphenyl) porphyrin (**c-PBe_2_(OH)_2_**)

**
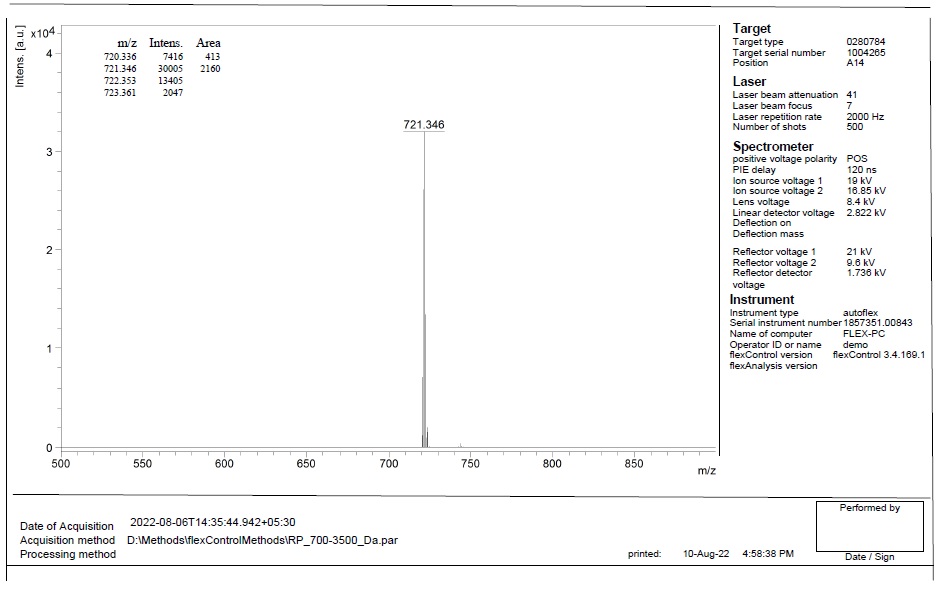
**
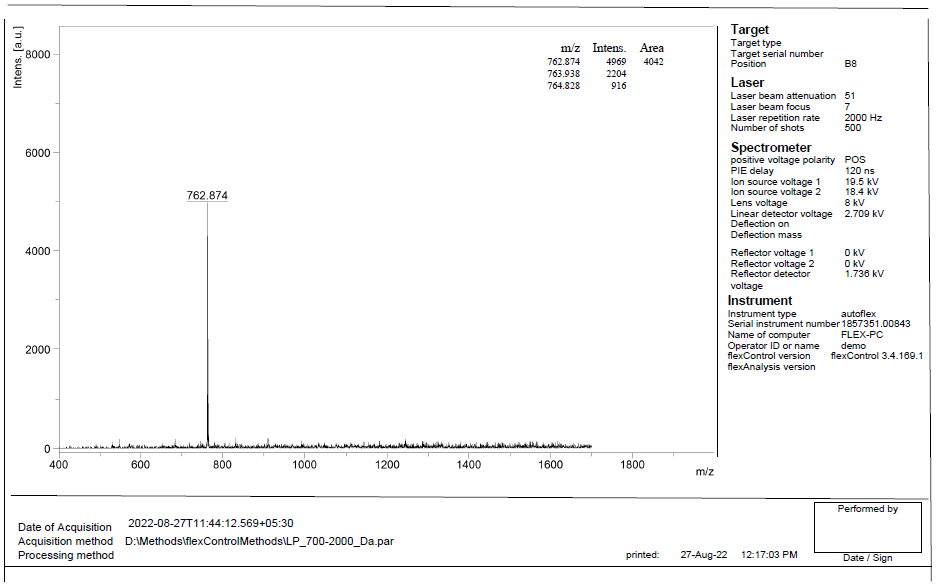
**Figure S15:** MALDI-TOF Spectra: 5,15-di-(4-methoxycarbonylphenyl)-10,20-(4-hydroxyphenyl) porphyrin (**t-PBe_2_(OH)_2_**)

**Figure S16:** MALDI-TOF Spectra: 5-(4-methoxycarbonylphenyl)-10,15,20-tri-(4-hydroxyphenyl) porphyrin (**PBe(OH)_3_**)


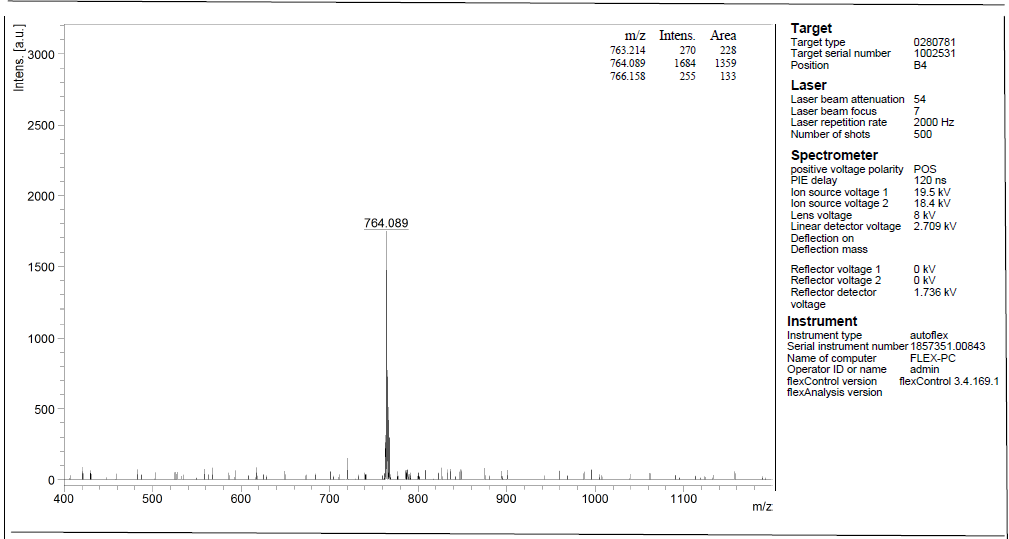
**Figure S17:** MALDI-TOF Spectra: 5,10,5-tri-(4-carboxyphenyl)-20-(4-hydroxyphenyl) porphyrin (**PB_3_(OH)**)

**
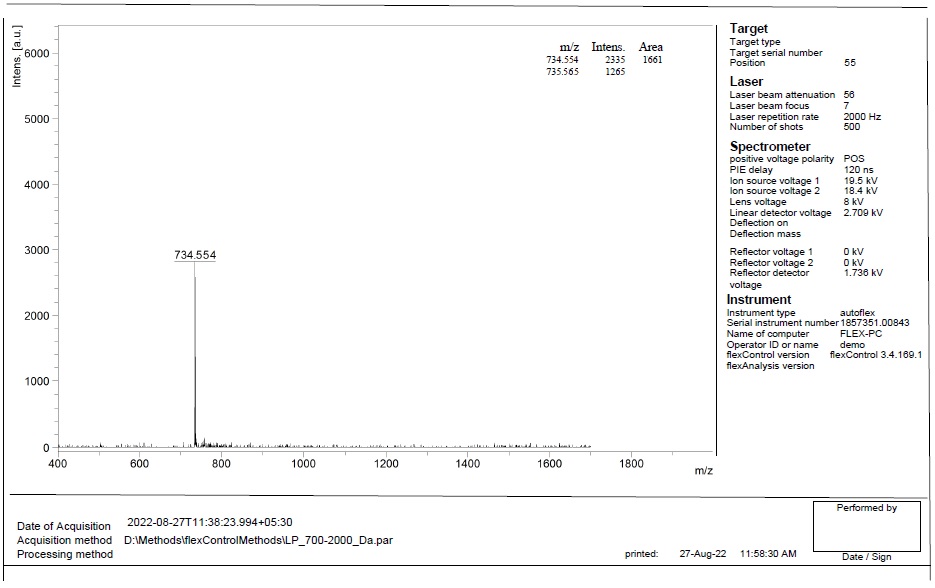
Figure S18:** MALDI-TOF Spectra: 5,10-di-(4-carboxyphenyl)-15,20-di-(4-hydroxyphenyl) porphyrin (**c-PB_2_(OH)_2_**)


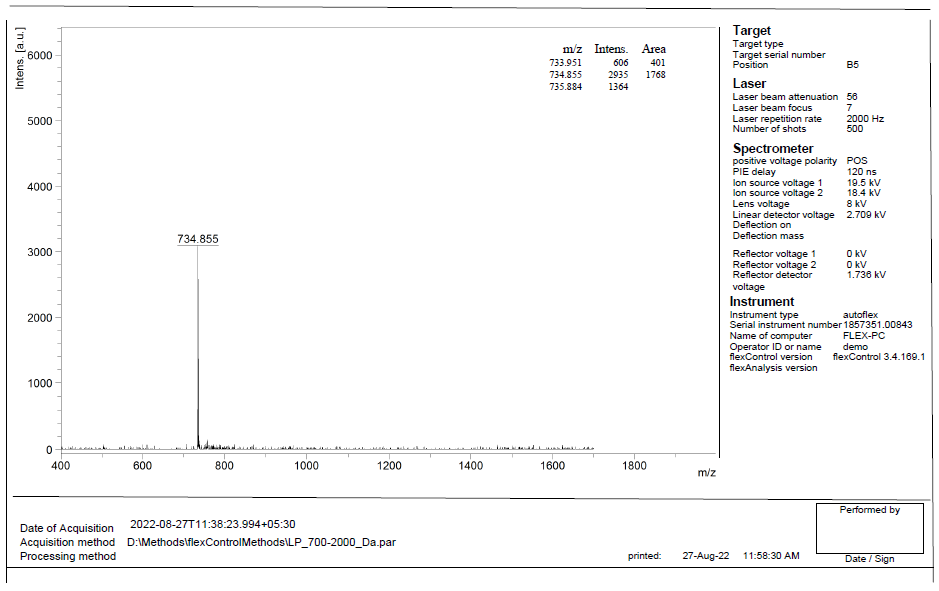
**Figure S19:** MALDI-TOF Spectra: 5,15-di-(4-carboxyphenyl)-10,20-di-(4-hydroxyphenyl) porphyrin (**t-PB_2_(OH)_2_**)


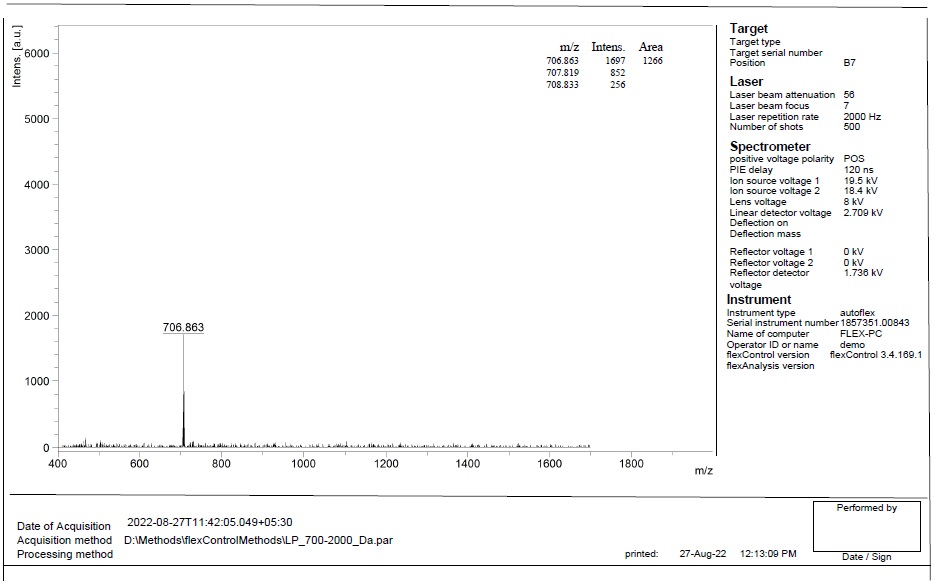


**Figure S20:** MALDI-TOF Spectra: 5-(4-carboxyphenyl)-10,15,20-tri-(4-hydroxyphenyl) porphyrin (**PB(OH)_3_**)


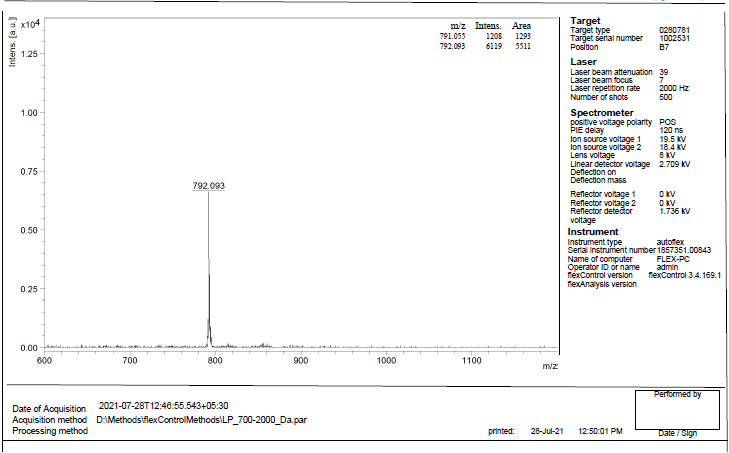
**Figure S21:** MALDI-TOF Spectra TCPP

**Table S5**. Cytotoxicity assay for selected cell lines in the presence of carboxyphenyl porphyrins under both non-PDT and PDT conditions. The CC_50_ value represents the mean ± standard deviations from three independent experiments.

| **Sl No.** | **Compounds** | **CC_50_ (under non-PDT conditions)** | | **CC_50_ (under PDT conditions)** |
| --- | --- | --- | --- | --- |
|  |  | **HEK293T cells (µM)** | **TZM-bl cells (µM)** | **TZM-bl cells (µM)** |
| 1. | **TCPP** | 52.152 | 50.867 | 2.712 |
| 2. | **THPP** | 50.534 | 54.915 | 3.139 |
| 3. | **PB_3_OH** | 54.820 | 56.658 | 4.899 |
| 4. | **c-PB_2_(OH)_2_** | 45.583 | 48.625 | 33.554 |
| 5. | **t-PB_2_(OH)_2_** | 47.585 | 49.790 | 35.250 |
| 6. | **PB(OH)_3_** | 51.642 | 56.367 | 32.841 |


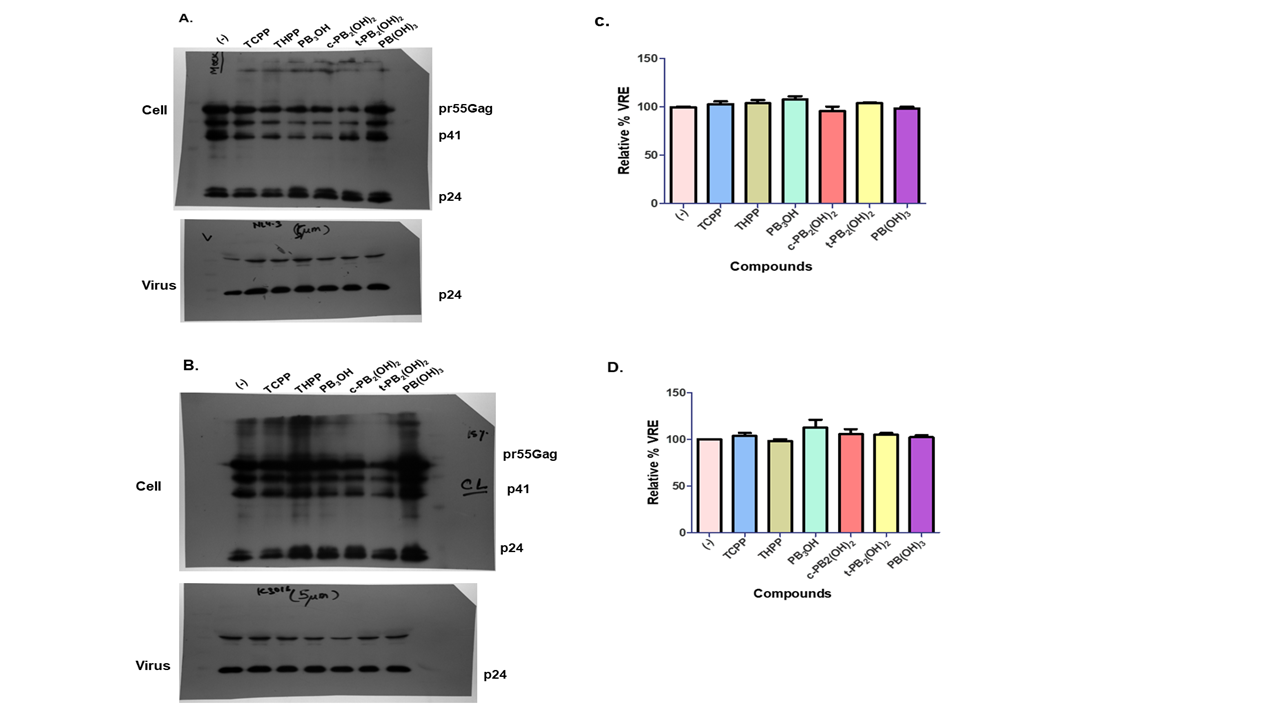


**Figure S22:** **Carboxyphenyl porphyrins did not affect HIV-1 Subtype B NL4-3 or subtype C K3016virus gene expression or release under non-PDT conditions.** Immunoblot analysis of virion and cell-associated **(a).** HIV-1 subtype B NL4-3 Gag and **(b).** HIV-1 subtype C K3016 Gag expression in the presence of carboxyphenyl porphyrins at 5 µM is shown. The position of HIV-1 p24, p41, and Pr55Gag is indicated (n=3). Percentage virus release efficiency (%VRE) of **(c).** HIV-1 subtype B NL4-3 virus and **(d).** HIV-1 subtype C K3016 virus released in the presence of the compound calculated as % of [virus-associated gag]/ [total (cell + virus-associated) Gag] relative to 5% DMSO in water (-) is shown(n=3).


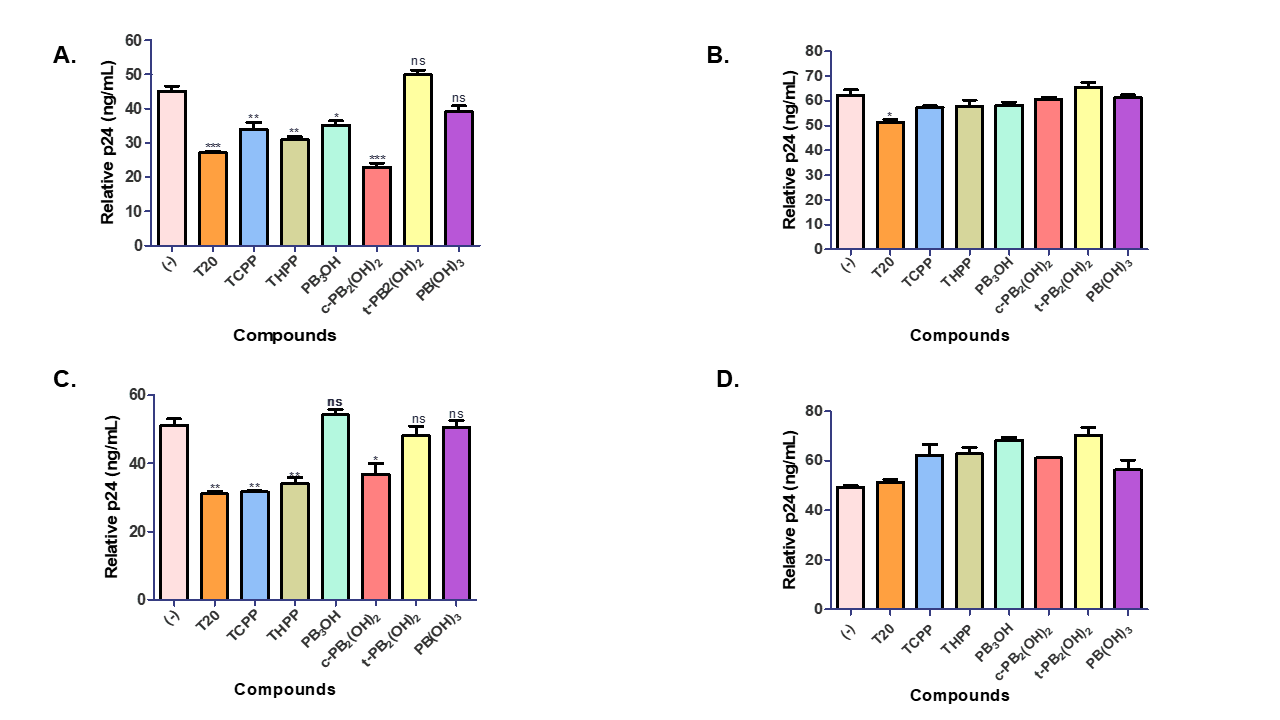


**Figure S23: Carboxyphenyl porphyrins prevented HIV-1 Subtype B NL4-3 or subtype C K3016 virus entry in T cells**—5 µM concentration of **(a).** Compounds were added during virus infection of HuTR5 cells with HIV-1 subtype B NL4-3 virus (n=3). **(b).** Compounds were added post-infection with HIV-1 subtype B NL4-3 virus (n=3). **(c).** Compounds were added during virus infection of HuTR5 cells with the HIV-1 subtype C K3016 virus (n=3).**(d).** Compounds were added post-infection with the HIV-1 subtype C K3016 virus (n=3). Relative p24 levels were quantified by ELISA on day 4 for HIV-1 subtype B NL4-3 virus and day 7 for HIV-1 subtype C K3016 virus and compared to 5% DMSO in water (-) control sample as shown (n=3). Enfuvirtide (T20) is an FDA-approved HIV-1 fusion inhibitor. Error bars indicate standard deviations. *p-value < 0.05, **p-value < 0.01, ***p value < 0.001: student’s t-test.


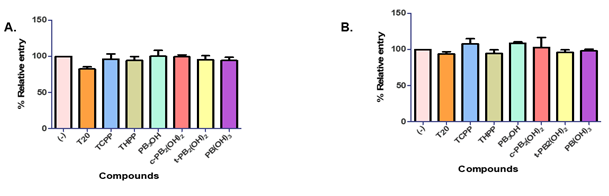


**Figure S24:** TZM-bl cells were pre-treated with 5 µM of carboxyphenyl porphyrins, 0.5µM T20, or 5% DMSO in water (-) for 2 h at 37 °C. Then, cells were washed and infected with 10 ng of HIV-1 p24 normalized. **(a).** HIV-1 Subtype B NL4-3 or **(b)**. K3016 virus for 2 h at 37 °C in the presence or absence of compounds. The effect of compounds on virus entry was estimated by measuring luciferase activity after 48h. Error bars indicate standard deviations from three independent experiments. *p-value <0.05**p-value <0.01***p value <0.001: student’s t-test.

Table S6

|  | Fluorescence Quantum Yield |
| --- | --- |
| Compound | Φ_F_ |
| PBe3OH | 0.067 |
| c-PBe_2_(OH)_2_ | 0.017 |
| t-PBe_2_(OH)_2_ | 0.166 |
| PBe(OH)_3_ | 0.028 |

References:

1. Checkley, M. A.; Luttge, B. G.; Mercredi, P. Y.; Kyere, S. K.; Donlan, J.; Murakami, T.; Summers, M. F.; Cocklin, S.; Freed, E. O., Reevaluation of the requirement for TIP47 in human immunodeficiency virus type 1 envelope glycoprotein incorporation. *J. Virol.* **2013,** *87* (6), 3561-3570.

2. Platt, E. J.; Wehrly, K.; Kuhmann, S. E.; Chesebro, B.; Kabat, D., Effects of CCR5 and CD4 cell surface concentrations on infections by macrophagetropic isolates of human immunodeficiency virus type 1. *J. Virol.* **1998,** *72* (4), 2855-2864.

3. Myśliwa-Kurdziel, B.; Solymosi, K.; Kruk, J.; Böddi, B.; Strzałka, K., Solvent effects on fluorescence properties of protochlorophyll and its derivatives with various porphyrin side chains. *Eur. Biophys. J.* **2008,** *37* (7), 1185-1193.

4. de Jong, M.; Seijo, L.; Meijerink, A.; Rabouw, F. T., Resolving the ambiguity in the relation between Stokes shift and Huang-Rhys parameter. *Phys. Chem. Chem. Phys.* **2015,** *17* (26), 16959-69.
